# A Plastic EMP1^+^ to LGR5^+^ Cell State Conversion as a Bypass to KRAS^G12D^ Pharmacologic Inhibition in Metastatic Colorectal Cancer

**DOI:** 10.1158/2159-8290.CD-25-0679

**Published:** 2025-10-21

**Authors:** Alessia Centonze, Adrià-Jaume Roura, Meritxell Novillo-Font, Cristina Giordano, Xavier Hernando-Momblona, Montserrat Llanses, Paula Prats, Marta Sevillano, Débora Cabot, Mireia Novell, Gabriel Pabst, Florian Andersch, Adrià Cañellas-Socias, Chong Zhang, Nikolaos-Nikiforos Giakoumakis, Hugh Sparks, Chris Dunsby, Julien Colombelli, Asunción Fernández-Barral, Elena Sancho, Camille Stephan-Otto Attolini, Alberto Muñoz, Antonio Barbachano, Héctor G. Palmer, Jordi Martínez-Quintanilla, Johannes Zuber, Cristina Blaj, Elsa Quintana, Carme Cortina, Marc A. Marti-Renom, Eduard Batlle

**Affiliations:** 1 Institute for Research in Biomedicine (IRB Barcelona), The Barcelona Institute of Science and Technology (BIST), Barcelona, Spain.; 2 Centro de Investigación Biomédica en Red de Cáncer (CIBERONC), Barcelona, Spain.; 3 Centre Nacional d’Anàlisi Genòmica, CNAG, Barcelona, Spain.; 4 Centre for Genomic Regulation, The Barcelona Institute for Science and Technology, Barcelona, Spain.; 5 Translational Program, Stem Cells and Cancer Laboratory, Vall d’Hebron Institute of Oncology (VHIO), Vall d’Hebron Barcelona Hospital Campus, Barcelona, Spain.; 6 Research Institute of Molecular Pathology (IMP), Vienna BioCenter (VBC), Vienna, Austria.; 7 Light Community, Department of Physics, Imperial College London, London, United Kingdom.; 8 Francis Crick Institute, London, United Kingdom.; 9 Instituto de Investigaciones Biomédicas Sols-Morreale, Consejo Superior de Investigaciones Científicas - Universidad Autónoma de Madrid, Madrid, Spain.; 10 Instituto de Investigación Sanitaria Hospital Universitario La Paz (IdiPAZ), Madrid, Spain.; 11 Revolution Medicines, Redwood City, California.; 12 Institució Catalana de Recerca i Estudis Avançats (ICREA), Barcelona, Spain.

## Abstract

Inhibitors of the oncogene KRAS hold promise for treating metastatic colorectal cancer (mCRC). In this study, we show that a selective, covalent small-molecule inhibitor of the active (ON) conformation of RAS-G12D, RMC-9945, exerts durable disease control in preclinical colorectal cancer models of early liver metastasis, but its therapeutic activity was diminished in the advanced metastatic disease. RMC-9945–treated metastases underwent a transition from a poor prognosis–associated *Emp1*^+^ transcriptional state to a WNT-driven *Lgr5*^+^ stem cell–like state that withstands the absence of RAS-G12D activity. This cell state change occurred within hours of RAS(ON) inhibitor treatment through a shift in transcription factor usage that involved limited chromatin remodeling. Forced conversion of metastatic cells to the *Lgr5*^+^ state through RAS-G12D inhibition, followed by genetic ablation of this population, reduced metastatic burden and prolonged survival in a mouse mCRC model. Overall, these preclinical findings demonstrate a central role for oncogenic KRAS in governing cellular plasticity in mCRC.

**Significance:**

We show that inhibition of oncogenic KRAS in preclinical models of advanced mCRC exerts a limited benefit, primarily due to the reversion of tumor cells to a stem cell–like state. Our findings highlight the context-dependent effects of oncogenic *KRAS* mutations and underscore cell plasticity as a therapeutic opportunity.

*See related commentary by Eng and Yilmaz et al., p. 201*

## Teaser

KRAS^G12D^ blockade in metastatic colorectal cancer drives transcriptional plasticity, shifting poor-prognosis EMP1^+^ cells into a WNT-activated LGR5^+^ cancer stem state that survives KRAS inhibition via AP-1 to TCF/LEF rewiring.

## Introduction

Metastatic colorectal cancer (mCRC) remains a major challenge in oncology due to limited therapeutic options. Recent advances in targeting the mutant *KRAS* oncogene represent a potentially transformative strategy for patients with few treatment alternatives (1), including those with *KRAS*-mutant mCRC. Approximately 40% of patients with colorectal cancer harbor activating mutations in *KRAS*, with the most common alterations—accounting for around 30% of cases—occurring at codon G12 (2–4). These mutations activate the MAPK signaling pathway, driving a wide array of protumorigenic processes, including proliferation, biosynthesis, invasion, survival, tumor microenvironment (TME) remodeling, and immunosuppression. The presence of mutant *KRAS* has been associated with metastatic disease and poor prognosis in colorectal cancer (5–7). However, clinical trials of small-molecule inhibitors targeting the inactive or GDP-bound state of the KRAS^G12C^ mutation (e.g., adagrasib) have shown a modest response rate of 19% in patients with colorectal cancer (8). A major mechanism of resistance to these KRAS^G12C^(OFF) inhibitors involves EGFR-mediated MAPK pathway reactivation (9). To overcome this mechanism, combination therapies including an anti-EGFR blocking antibody—such as adagrasib with cetuximab—have been evaluated, resulting in an increased response rate of 46%, though the median duration of response only improved from 4.3 to 7.6 months (8). These findings underscore the urgent need to better understand resistance mechanisms to KRAS inhibition in colorectal cancer to enhance therapeutic efficacy.

Colorectal cancers are composed of heterogeneous populations of tumor cells exhibiting distinct transcriptional states, but only a small proportion of this transcriptomic diversity can be attributed to clonal and subclonal genetic alterations (10). Instead, intratumoral heterogeneity largely stems from the plasticity of cancer cells; i.e., their ability to adopt different transcriptional states without permanent genetic changes (10). It has been well established that much of the phenotypic and functional heterogeneity in colorectal cancer recapitulates the homeostatic renewal dynamics of the colonic epithelium (reviewed in ref. 11). The mutational activation of WNT signaling that occurs at the onset of tumorigenesis drives a gene expression program reminiscent of healthy intestinal stem cells (ISC) in most colorectal cancers (11). Elevated levels of this ISC-like transcriptional program define cancer stem cells (CSC), which can be recognized by the expression of canonical stem cell genes such as *LGR5* (11). *LGR5*^+^ CSCs isolated from patient colorectal cancer samples can efficiently initiate tumors when transplanted into recipient mice whereas their differentiated progeny cannot (11). In genetic mouse models of colorectal cancer, *Lgr5*^+^ CSCs sustain metastatic outgrowth (11–13).

Despite the extensive evidence supporting a key role for *LGR5*^+^ CSCs in colorectal cancer, several lines of evidence, including our own, revealed a critical role for *Lgr5*-negative tumor cell populations in therapy resistance and metastatic dissemination (13–23). Our analyses of patient-derived colorectal cancer samples showed heterogeneous mixtures of *LGR5*^+^ CSCs and *LGR5*-negative cells with distinct transcriptional identities (13). Notably, we identified a specific subset of *LGR5*-negative cells expressing a gene signature associated with a high risk of metastatic recurrence following surgery of the primary tumor (13). We termed this oncofetal-like population high-relapse cells (HRC). HRCs are characterized by elevated expression of epithelial membrane protein 1 (*EMP1*), are abundant in stages III and IV colorectal cancers, express mediators of collective cell migration, and localize to the invasive fronts of primary tumors in both human and mouse mCRC models (13). In mouse colorectal cancer models, disseminated *Emp1*^+^ HRCs colonize the liver and, through transcriptional plasticity, regenerate the *Lgr5*^+^ CSC population (13). Genetic ablation of *Emp1*^+^ HRCs prevents metastatic relapse after surgery of the primary colorectal cancer, demonstrating that the acquisition of the HRC state is necessary for metastasis in preclinical models (13). Additional studies have shown that chemotherapy can induce the plastic transition of *LGR5*^+^ CSCs into a *LGR5*-negative state characterized by activation of fetal gut and regenerative gene programs (15–17). Remarkably, a recent study found that metastatic lesions of patients with colorectal cancer exhibit elevated transcriptional plasticity and are enriched in *L1CAM*^+^/*EMP1*^+^ tumor cells compared with primary colorectal cancers. This cell population expresses the HRC/fetal gut–like gene program while suppressing the intestinal *LGR5*^+^ ISC program (18). Collectively, these and other studies reveal that tumor cell plasticity is pervasive in colorectal cancer and that the *LGR5*^+^ and *EMP1*^+^ cell states are not fixed but dynamically regulated by signals emanating from the TME or induced by therapy (24, 25). However, the role of genetic alterations in oncogenes and tumor suppressors in modulating tumor cell plasticity remains largely unexplored.

RMC-9945 is a RAS(ON) G12D-selective, covalent inhibitor and preclinical tool compound representative of the investigational agent zoldonrasib (RMC-9805) that is currently being evaluated in clinical trials (26). Like zoldonrasib, RMC-9945 forms a tri-complex between GTP-bound, active RAS G12D and the abundant intracellular chaperone cyclophilin A (26). This binary complex then selectively and rapidly covalently modifies Asp12 of RAS-G12D, leading to inhibition of RAS effector binding and downstream signaling (26). Zoldonrasib elicited robust and durable responses in patient-derived xenograft (PDX) models of pancreatic adenocarcinoma (PDAC), non–small cell lung cancer (NSCLC) and gastric adenocarcinoma. In contrast, the authors reported a low response rate in colorectal cancer PDX and cell line–derived xenograft models (26). In this study, we investigated the therapeutic activity of RMC-9945 in preclinical models of mCRC and discovered a key role for oncogenic KRAS in promoting CSC-to-HRC cell state transitions.

## Results

### KRAS^G12D^ Inhibition Enforces a *Lgr5*^+^ ISC-like Program in mCRC

**Figure 1.**
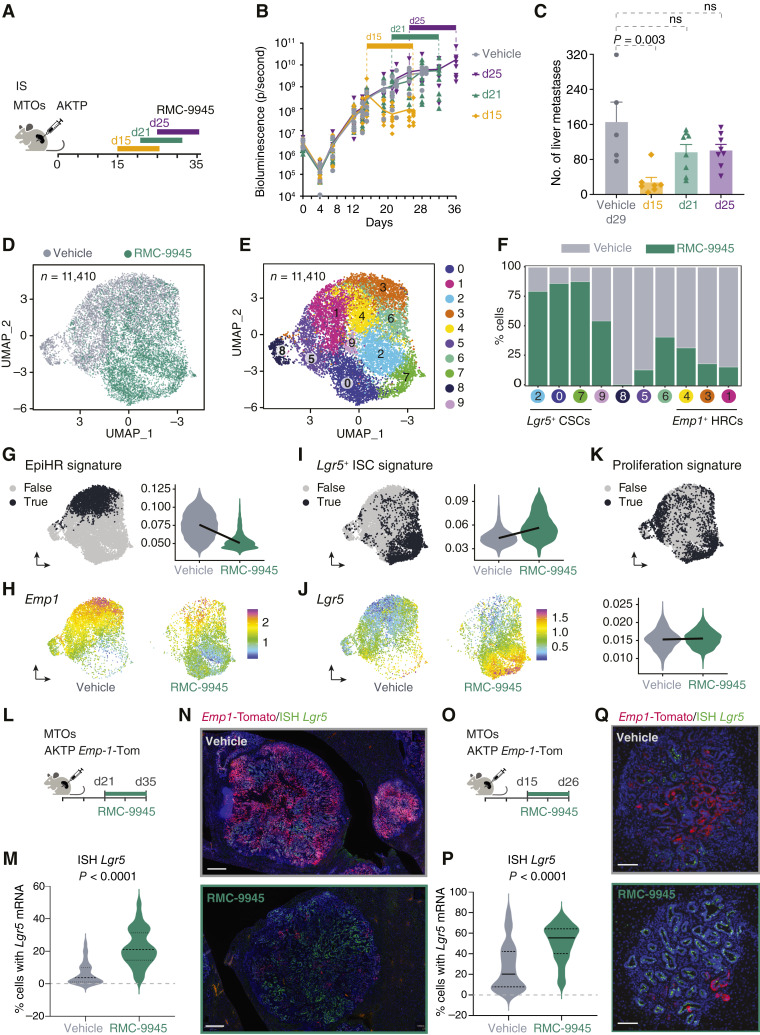
KRAS^G12D^ inhibition enforces a *Lgr5*^+^ ISC-like transcriptional program in liver metastases. **A,** Experimental setup of RMC-9945 treatment of liver metastases. Treatment started at day 15, 21, or 25 after inoculation of liver metastases implanted in the spleen (IS) of C57BL/6J mice and continued for 11 days. **B,** Longitudinal intravital bioluminescence quantification of AKTP MTOs treated with vehicle or RMC-9945. **C,** Treatment from day 15, day 21, and day 25 of established liver metastases (LiM) generated by injecting AKTP MTOs in the spleen of C57BL/6J mice. Number of liver metastases. Vehicle *n* = 5 mice, D15 *n* = 7, D21 *n* = 8, and D25 = 8. Mean ± SEM; *P* values are derived from ANOVA followed by two-sided Dunnett tests. **D,** UMAP visualization of scRNA-seq (*n* = 11,410) showing vehicle-treated (gray) and RMC-9945–treated (green) cells. Mice were treated from day 21 to day 26 (6 days), and liver metastasis dissected. **E,** UMAP projection with data points colored by Louvain clusters (resolution 0.6), in which each cluster is represented in a unique color to emphasize the dataset’s underlying structure. **F,** Bar plot showing the frequency distribution of cells in the vehicle and RMC-9945 groups within each cluster, ordered by their association with CSC, intermediate, or HRC states. Percentages represent the proportion of cells in each group per cluster. **G** and **H,** UMAP visualization and violin plot showing HRC (EpiHR^+^) cells (**G**) and the expression (MAGIC values) of the *Emp1* gene (**H**). **I** and **J,** UMAP visualization and violin plot showing CSCs (*Lgr5*^+^ ISC-like) and the expression (MAGIC values) of the *Lgr5* gene (**J**). MAGIC, Markov Affinity-based Graph Imputation of Cells. **K,** UMAP visualization and violin plot showing proliferative cells. Gene signatures used for the analysis are detailed in Supplementary Table S1. In the UMAP visualization, black indicates the top 20% (quantile 80) of cells based on feature expression (true) and gray indicates the remaining cells (false). The gene expression UMAP is split by the vehicle and RMC-9945 groups. **L,** Treatment from day 21 of established liver metastases (LiM) generated by injecting AKTP Emp1-Tom MTOs in the spleen of C57BL/6J mice. **M,** Percentage of *Lgr5* mRNA spots detected by RNAscope on the total cell number detected in each metastasis. Each dot represents one metastasis. Vehicle *n* = 56, RMC-9945 *n* = 71. Mean ± SEM; *P* values are derived from unpaired *t* test. **N,** Representative example of *Lgr5* mRNA FISH combined with tdTomato immunofluorescence on liver metastases sections. Scale bar, 400 μm. **O,** Treatment from day 15 of established liver metastases (LiM) generated by injecting AKTP MTOs Emp1-Tom in the spleen of C57BL/6J mice. **P,** Percentage of *Lgr5* mRNA spots detected by RNAscope on the total cell number detected in each metastasis. Each dot represents one metastasis. Vehicle *n* = 95, RMC-9945 *n* = 87. Mean ± SEM; *P* values are derived from unpaired *t* test. **Q,** Representative example of *Lgr5* mRNA FISH combined with tdTomato immunofluorescence on liver metastases sections. Scale bar, 100 μm.

We have previously described mouse tumor organoids (MTO; refs. 13, 27). They were established from colorectal cancers that arose in genetically engineered mouse models bearing loss-of-function alleles in *Apc*, *Trp53*, and *Tgfbr2* plus a *Kras*^G12D^ gain-of-function allele (AKTP MTOs; refs. 13, 27). Inoculation of AKTP MTOs through the portal vein of syngeneic c57/Bl6 mice generates a model of aggressive human-like *KRAS* mutant liver metastatic disease (13). We found that the antitumor activity of the RAS(ON) G12D-selective inhibitor RMC-9945 depended on the day treatment started after liver metastases were established (Fig. 1A–C; Supplementary Fig. S1A–S1E). Daily oral administration of RMC-9945 at 100 mg/kg exerted antitumor activity when treatment began at day 15 after MTO inoculation. In this setting, there was a reduction in both the number and size of liver metastatic nodules (Fig. 1A–C; Supplementary Fig. S1B and S1E). Nevertheless, many metastases in the early setting were not eliminated but persisted as drug-tolerant lesions throughout the duration of RMC-9945 treatment (Supplementary Fig. S1B). When treatment was initiated later, either on day 21 or 25 after inoculation, RMC-9945 exerted a limited therapeutic response. Metastases continued growing during treatment (Fig. 1B), and the reduction in metastases burden was not significant (Fig. 1C; Supplementary Fig. S1C–S1E).

We initially focused on elucidating the mechanisms of resistance to KRAS^G12D^ inhibition in the late therapeutic paradigm, i.e., treatment initiation on day 21, and characterized the effect of RMC-9945 using single-cell RNA sequencing (scRNA-seq). Figure 1D–F and Supplementary Figs. S2A–S2E, S3A, S3B and S4A, S4B describe transcriptional cell states based on the expression of well-established gene signatures and marker genes. KRAS^G12D^ inhibition produced remarkable transcriptional reprogramming, with cell states showing limited overlapping between vehicle-treated (control) and RMC-9945–treated metastases (Fig. 1D–F). Vehicle-treated metastases exhibited abundance of HRCs indicated by the expression of the poor prognosis EpiHR signature (Fig. 1G; Supplementary Figs. S2A, S2B and S3A; Supplementary Table S1) and the marker gene *Emp1* (Fig. 1H; Supplementary Fig. S3B; ref. 13). These cells also expressed the signature of fetal gut organoids (Supplementary Fig. S2A and S2B; ref. 28). *Emp1*, *Anxa2*, *Msln*, *Plaur*, and other well-established marker genes of fetal-like cells and HRCs were expressed at varying levels across clusters 1, 4, and 7, highlighting heterogeneity of cell states within the poor prognosis-associated tumor cell population (Supplementary Fig. S3B). In addition, a small subset of metastatic cells in control mice (cluster 8) underwent intestinal epithelial differentiation shown by elevated expression of *Krt20* and of the enterocyte gene program (Supplementary Fig. S2A and S2D).

All these transcriptomic cell states were largely depleted upon treatment with the RAS(ON) G12D-selective inhibitor RMC-9945 (Fig. 1D and F). Instead, KRAS^G12D^–inhibited metastases were mainly formed by tumor cells that upregulate the WNT-driven *Lgr5*^+^ ISC gene signature (Fig. 1I; Supplementary Figs. S2A, S2C and S4; Supplementary Table S1; ref. 29). A detailed analysis showed that a large proportion of canonical *Lgr5*^+^ ISC program (29) was induced by RMC-9945 in cell clusters 0, 2, and 7 (Fig. 1F; Supplementary Fig. S4A), including well-established ISC marker genes such as *Lgr5* (Fig. 1J), *Ascl2*, *Smoc2*, *Axin2*, *or Notum* (Supplementary Figure S4B). *Lgr5* and *Axin2* were most highly expressed in cluster 7, whereas *Notum*, another stem cell marker gene, was particularly upregulated in cluster 2. Therefore, RMC-9945 induces overlapping but distinct WNT-activated states in liver metastases (Supplementary Figs. S2A and S4). For simplicity, we will refer the tumor cells in cluster 2, 0, and 7 as *Lgr5*^+^ CSCs. Proliferation was driven by distinct cell subsets in vehicle-treated and RMC-9945–treated metastases (Fig. 1K; Supplementary Fig. S2A and S2E), with marginal changes in the number of cells or expression levels of proliferation-related genes between the two conditions. To strengthen these findings, we generated liver metastases by inoculating AKTP MTOs engineered with a TdTomato cassette integrated into the *Emp1* locus that labels HRCs (13) and at the same time detected *Lgr5* mRNA expression by RNAscope (Fig. 1L–N). This experiment corroborated a prominent conversion of metastatic cells from the *Emp1*^+^ HRC to an *Lgr5*^+^ CSC state upon inhibition of KRAS^G12D^ activity by RMC-9945 treatment (Fig. 1L–N). Importantly, RMC-9945 treatment also significantly increased the number of *Lgr5*^*+*^ cells when treatment started at day 15 after inoculation, suggesting that an equivalent plasticity mechanism is elicited by KRAS^G12D^ inhibition in early versus late metastases (Fig. 1O–Q). Finally, we quantified the number of HRCs during metastatic progression. We found that late metastases have more *Emp1*^+^ HRCs than early metastases (Supplementary Fig. S4C). Therefore, the growth suppression effect observed in the early treatment setting cannot be attributed to targeting a higher proportion of *Emp1*^+^ HRCs.

### Plasticity from *Emp1*^+^ HRC to *Lgr5*^+^ CSC States by Oncogenic KRAS and BRAF Inhibition across Colorectal Cancer Models

**Figure 2.**
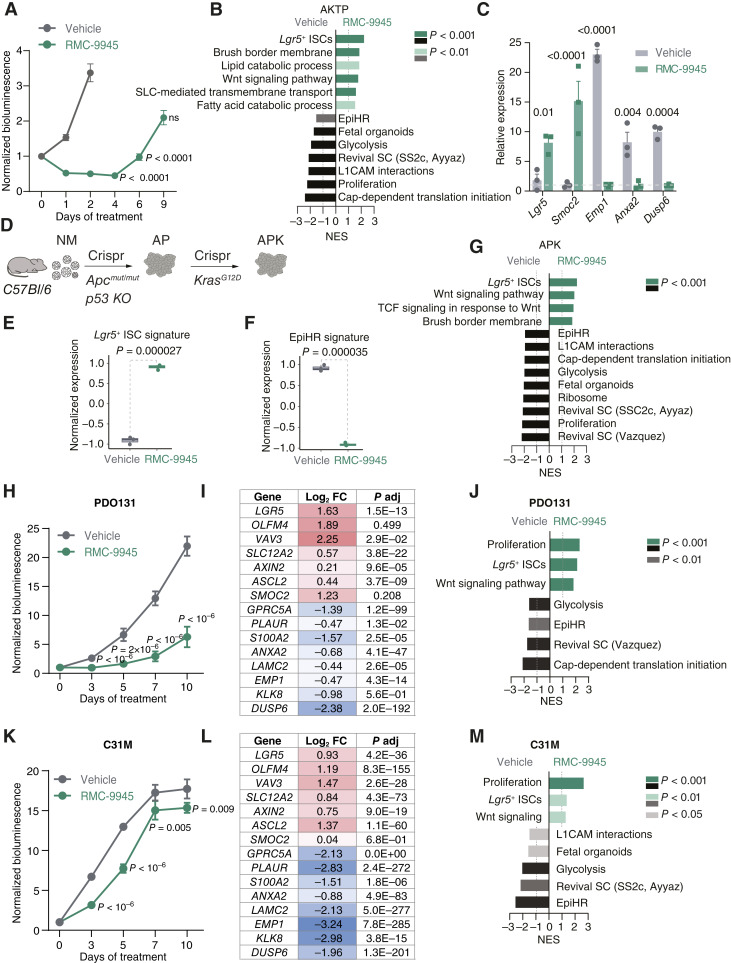
Plasticity between *Lgr5*^+^ CSC to *Emp1*^+^ HRC states by KRAS activity across models. **A,** Growth (CellTiter-Glo) of AKTP MTOs treated with vehicle or RMC-9945 for 9 days normalized to day 0 of treatment. Mean ± SEM. *P* values between vehicle day 2 and RMC-9945 day 4, 6, or 9 are derived from ANOVA followed by Šídák multiple comparisons test. **B,** Gene set enrichment analysis of AKTP MTOs upon vehicle versus RMC-9945 treatment. **C,** Relative mRNA expression of indicated marker genes in vehicle-treated and RMC-9945–treated AKTP MTOs for 48 hours assessed by RT-qPCR. Mean ± SEM. *P* values are derived from ANOVA followed by Šídák multiple comparisons test. **D,** CRISPR-Cas9 targeting strategy to introduce APK mutations in normal mucosa (NM) organoids derived from C57BL/6J mice. **E** and **F,** Boxplots depicting the normalized and z-scored expression of *Lgr5*^+^ ISC gene signature (**E**) and EpiHR signature (**F**) in APK CTOs treated with either vehicle or RMC-9945 for 48 hours. *t* test was used to assess median differences. **G,** Gene set enrichment analysis (GSEA) of APK CTOs upon vehicle and RMC-9945 treatment. **H,** Growth (CellTiter-Glo) of PDO131 treated with vehicle or RMC-9945 for 10 days normalized to day 0 of treatment. **I,** Differential gene expression analysis of CSC and HRC key genes for PDO 131. **J,** GSEA of PDO131 in vehicle versus RMC-9945 treatment. **K,** Growth (CellTiter-Glo) of PDO C31M treated with vehicle or RMC-9945 for 10 days normalized to day 0 of treatment. **L,** Differential gene expression analysis of CSC and HRC key genes for PDO C31M. **M,** GSEA of PDO C31M in vehicle versus RMC-9945 treatment. PDOs were treated with RMC-9945 for 4 days and subjected to RNA-seq analysis. *Dusp6* gene is included as a reference for KRAS/MAPK pathway inhibition. Log_2_ fold change (FC) and adjusted *P* value are shown. For GSEA, selected Hallmark, GOCC, Kyoto Encyclopedia of Genes and Genomes, and custom gene signatures are shown. NES, normalized enrichment score. *P* value < 0.05 for all gene sets, color gradient represents significance as shown. For the growth curves, *P* values are derived from multiple unpaired *t* test.

We next assessed the generality of the cell state conversion triggered by KRAS inhibition using colorectal cancer organoid models (Fig. 2A–G; Supplementary Figs. S5A–S5C, S6A–S6J and S7A–S7F; Supplementary Table S2). Treatment with the RAS(ON) G12D-selective inhibitor RMC-9945 initially suppressed the growth of AKTP MTOs *in vitro*; however, after this initial phase, the MTOs resumed growth in the presence of the compound (Fig. 2A). RNA-seq confirmed that RMC-9945 induced the upregulation of a substantial proportion of the *Lgr5*^+^ ISC program whereas it downregulated the expression of the EpiHR poor prognosis gene set, as well as several other signatures characteristic of HRCs, such as fetal gut–like or regenerative stem cell gene signatures (Fig. 2B; Supplementary Fig. S5A–S5C; refs. 28, 30). RT-qPCR analysis confirmed a significant increase in ISC marker genes *Lgr5* and *Smoc2*, along with a downregulation of HRC markers *Emp1* and *Anxa2* (Fig. 2C). As expected, the levels of *Dusp6*, a gene activated by MAPK signaling downstream of KRAS in multiple tumor types (31, 32), was also downregulated by RMC-9945 (Fig. 2C). The effect of oncogenic KRAS inhibition in promoting plasticity was also evident upon treatment with MRTX1133, a small-molecule inhibitor targeting the GDP-bound configuration of KRAS^G12D^ (Supplementary Fig. S6A–S6C; Supplementary Table S3; refs. 33, 34). To further explore the connection between compound resistance and acquisition of the CSC *Lgr5*^*+*^ state, we used AKTP MTOs with *Lgr5*-EGFP and *Emp1*-TdTomato reporter knock-in alleles (13). Tracking of fluorescence confirmed that during the regrowth phase (d4–d7 of treatment), AKTP MTOs were composed of *Lgr5*-EGFP-bright cells (Supplementary Fig. S7A–S7E). However, we noticed lower *Lgr5*-EGFP reporter levels at day 9. There was also a progressive increase in *Dusp6* expression over time in the presence of RMC-9945, suggestive of MAPK reactivation, which coincided with decreased expression of CSC genes and upregulation of HRC markers during the relapse phase (Supplementary Fig. S7F). These observations imply that over a prolonged *in vitro* treatment, AKTP MTOs progressively develop resistance to RMC-9945 and reactivate oncogenic KRAS signaling (Supplementary Fig. S7).

We also studied the effect of the RAS(ON) G12D-selective inhibitor RMC-9945 on CRISPR-generated MTOs (CTOs; Fig. 2D–G). These models were engineered by introducing colorectal cancer driver mutations into organoids derived from healthy colonic mucosa through CRISPR-mediated mutagenesis (Fig. 2D; refs. 13, 35). RNA-seq of isogenic *Apc* and *Trp53* (AP) versus *Apc*, *Trp53*, and *Kras*^G12D^ (APK) CTOs showed that introduction of *Kras*^G12D^ allele was sufficient to upregulate the poor prognosis EpiHR signature and downregulate the *Lgr5*^+^ CSC program (Supplementary Fig. S6D–S6F). Global gene expression analysis by RNA-seq confirmed the overall downregulation of the EpiHR signature and upregulation of the *Lgr5*^+^ ISC program in APK CTOs treated with RMC-9945 (Fig. 2E and F). Gene sets related to WNT/TCF signaling, brush border formation, proliferation, glycolysis, and ribosomal biosynthesis were also consistently regulated by KRAS activity in both MTO and CTO models (Fig. 2B and G; Supplementary Table S2). In contrast, RMC-9945 exerted only a minor effect on AP CTOs, which is consistent with the lower inhibitory activity of the small molecule against the wild-type (WT) KRAS protein (Supplementary Fig. S6G and S6H; ref. 26). The KRAS^G12D^ inhibitor MRTX1133 produced similar effects to RMC-9945 on the APK CTO model (Supplementary Fig. S6I and S6J; Supplementary Table S3).

We next assessed the effects of RMC-9945 on human colorectal cancer models. In these experiments, we used two patient-derived organoids (PDO) bearing KRAS^G12D^ mutation derived from different patients (genotypes in Supplementary Table S4). PDO131 was derived from a primary colorectal cancer and C31M from a liver metastasis (Fig. 2H–M). PDO131 continued to expand under RMC-9945 treatment, albeit at a reduced rate, whereas C31M was largely resistant to the compound’s growth-suppressive effects (Fig. 2H and K). RNA-seq revealed consistent upregulation of the *Lgr5*^+^ ISC program and downregulation of the EpiHR signature in the two PDO models after treatment with the RAS(ON) inhibitor (Fig. 2I and L). Gene set enrichment analysis analysis confirmed regulation of similar gene programs by RMC-9945 in the two KRAS^G12D^ PDO models, including increased expression of WNT/TCF-related genes (Fig. 2J and M; Supplementary Table S2).

We conclude that for several mouse (MTOs and CTOs) and human (PDOs) colorectal cancer model systems, KRAS-activating mutations promote the EMP1^+^ HRC state while suppressing the default LGR5^+^ CSC program imposed by constitutive activation of the WNT pathway by mutations. Pharmacologic KRAS^G12D^ inhibition reverses this transcriptional switch.

### Plasticity Induced by BRAF^V600E^ Inhibition

We have previously shown that the poor prognosis EpiHR signature also correlates with *BRAF* mutation in the colorectal cancer dataset of The Cancer Genome Atlas (TCGA; ref. 13). Encorafenib is a BRAF^V600E^-selective inhibitor typically combined with the anti-EGFR mAb cetuximab for the treatment of mCRC (36). We established PDX organoids (PDXO) from two *BRAF*^*V600E*^-mutant liver metastases from patients who had progressed on BRAF inhibitor therapy and tested the treatment effects in these models (Supplementary Fig. S8A–S8D). Although encorafenib plus cetuximab slowed PDXO growth, both models ultimately expanded under treatment (Supplementary Fig. S8A and S8C). Gene expression profiling revealed that encorafenib reduced expression of HRC marker genes while upregulating the *LGR5*^+^ CSC signature (Supplementary Fig. S8B and S8D). Therefore, the *EMP1*^+^ HRC transcriptional state is maintained by either constitutive BRAF or KRAS signaling activity in colorectal cancer. Pharmacologic inhibition of these oncogenes enforces a plastic transition toward the *LGR5*^+^ CSC state.

### PDAC and Keratinization-Associated Genes Induced by KRAS Activating Mutations

**Figure 3.**
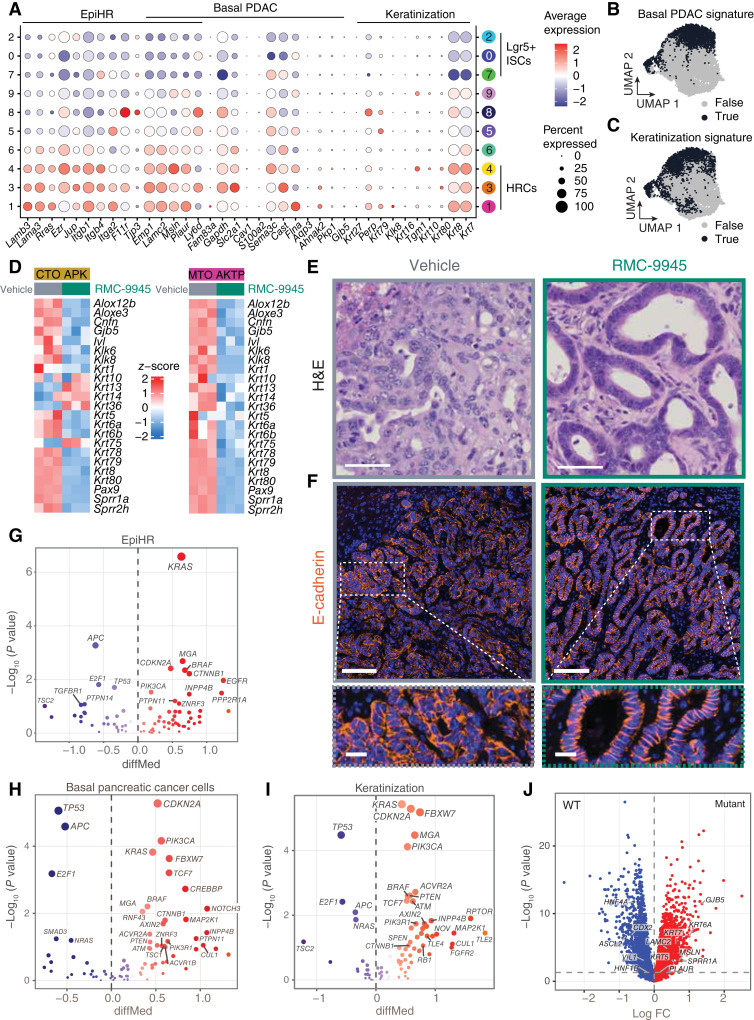
PDAC and keratinization gene programs induced by KRAS activating mutations. **A,** Dot plot illustrating the scaled and normalized expression of selected marker genes across the different metastasis cell clusters (Louvain clustering, resolution = 0.6, SCT values). **B,** UMAP visualization showing PDAC basal cells (**B**) and epidermal keratinization (**C**) with black indicating the top 20% (quantile 80) of cells based on feature expression (TRUE) and gray indicating the remaining cells (FALSE). **D,** Heatmaps of in vitro APK CTOs and AKTP MTOs treated with vehicle or RMC-9945 for 48 hours, hierarchically clustered using the Ward D2 method based on keratinization gene list. **E,** Representative example of hematoxylin and eosin (H&E) staining on liver metastases treated with vehicle or RMC-9945 for 11 days. Scale bar, 50 μm. **F,** Representative example of E-cadherin immunofluorescence on liver metastases treated with vehicle or RMC-9945. Top scale bar, 100 μm. Bottom scale bar, 20 μm. **G–I,** Volcano plot showing expression differences of the EpiHR signature (**G**), the basal pancreatic signature (**H**), and keratinization genes (**I**) between samples with and without driver mutations in individual oncogenes and tumor-suppressor genes from the COAD TCGA patient cohort (*n* = 520 patients). The difference of median values (x axis) versus *P* values (Wilcoxon rank-sum test; y axis) are shown. **J,** Volcano plot of differential expression of samples with and without mutations in the KRAS signaling pathway. Genes of interest are highlighted. FC, fold change.

As detailed in the introduction, EMP1^+^ HRCs cells were originally identified by expression of a poor prognosis gene signature (EpiHR) that predicts metastatic relapse in American Joint Committee on Cancer stage I–III patients with colorectal cancer (13). This malignant cell state is featured by the upregulation of genes encoding hemidesmosome components (*Lamb3*, *Lama3*, and *Lamc2*), actin cytoskeleton organizers (*Rras*, *Ezr*, and *Rhof*), extracellular matrix attachment molecules (*Itga2*, *Itgb1*, and *Itgb4*), and proteins involved in tight junction formation (*Emp1*, *Tjp3*, *Jup*, and *F11r*; Fig. 3A; ref. 13). We previously showed that KRAS-activated HRCs also express several marker genes of the basal PDAC subtype such as *Lamc2*, *Msln*, or *Plaur* (Fig. 3A and B; refs. 13, 37). A recent study revealed exacerbated plasticity in colorectal cancer patient metastases, characterized by the loss of the intestinal gene programs and the emergence of a fraction of cells undergoing squamous epithelium-like differentiation (18) and loss-of-function mutations in the chromatin remodeling factor ATRX contribute to this effect (38). A squamous esophageal cancer program is also present in oncofetal cells of colorectal cancer organoid models (19). Indeed, further inspection of the scRNA-seq data revealed that a subset of *Emp1*^+^ HRCs exhibit elevated levels of several keratin encoding genes and keratinization mediators (*Krt27*, *Krt79*, *Krt80*, *Klk8*,*Perp*, and *Tgm1*) that are typically expressed in squamous epithelia but not in the gastrointestinal epithelium (Fig. 3A and C). Furthermore, KRAS^G12D^ drove the expression of multiple squamous keratinization genes in MTO and CTO models, as revealed by downregulation upon RMC-9945 supplementation (Fig. 3D).

We also noticed that metastases treated with RMC-9945 gained evident glandular organization, with tumor cells exhibiting apico-basal polarization reminiscent of that of the colonic epithelium (Fig. 3E). This repolarization did not correlate with increased E-cadherin or other cell-to-cell adhesion molecules at the mRNA level (Supplementary Fig. S9A). However, in control metastases, we observed a diffuse cytoplasmic accumulation of E-cadherin, indicative of impaired adherens junction formation and cell polarization, in contrast to the sharp basolateral localization seen after RMC-9945 treatment (Fig. 3F).

To assess the relevance of these findings to human colorectal cancer, we analyzed the TCGA colorectal cancer genomic and transcriptomic dataset (3). The poor prognosis (EpiHR) signature of *EMP1*^+^ HRCs was significantly upregulated in patients with colorectal cancer bearing MAPK signaling pathway activating mutations and more prominently oncogenic *KRAS* mutations (Fig. 3G). Further analyses confirmed that acquisition of *KRAS* activating mutations correlate with expression of the basal PDAC (Fig. 3H) and keratinization gene signatures (Fig. 3I). Differential gene expression analysis also showed that several marker genes of PDAC and keratinization were upregulated in the MAPK-mutant pathway versus WT colorectal cancers in the TGCA cohort (Fig. 3J). Among them, *KRT5* and *KRT6A* are expressed in basal cells of the skin, esophagus, and lung epithelium and they also feature basal and squamous PDAC subtypes (39). Conversely, the levels of intestinal epithelium-defining transcription factors (TF) *CDX2*, *ASCL2*, and *HNF4A* were significantly lower in patients with colorectal cancer bearing activating mutations in MAPK pathway components (Fig. 3J). Treatment of PDO models with RMC-9945 reduced the expression of basal keratins and basal PDAC genes, this effect being more prominent in the metastasis-derived PDO C31M (Supplementary Fig. S9B–S9D). We also observed that RMC-9945 increased the levels of the intestine-defining TFs *CDX2*, *CDX1*, *ASCL2*, and *HNF4A* in APK CTOs (Supplementary Fig. S9E), AKTP MTOs (Supplementary Fig. S9F), and the PDO C31M (Supplementary Fig. S9B). Overall, these data support that the activity of KRAS^G12D^ facilitates the expression of poor prognosis–associated nonintestinal genes in colorectal cancer cells whereas RAS(ON) G12D-selective inhibitor RMC-9945 reestablishes an intestinal epithelial-like identity.

### Dynamics of Cell State Conversion by KRAS Inhibition

**Figure 4.**
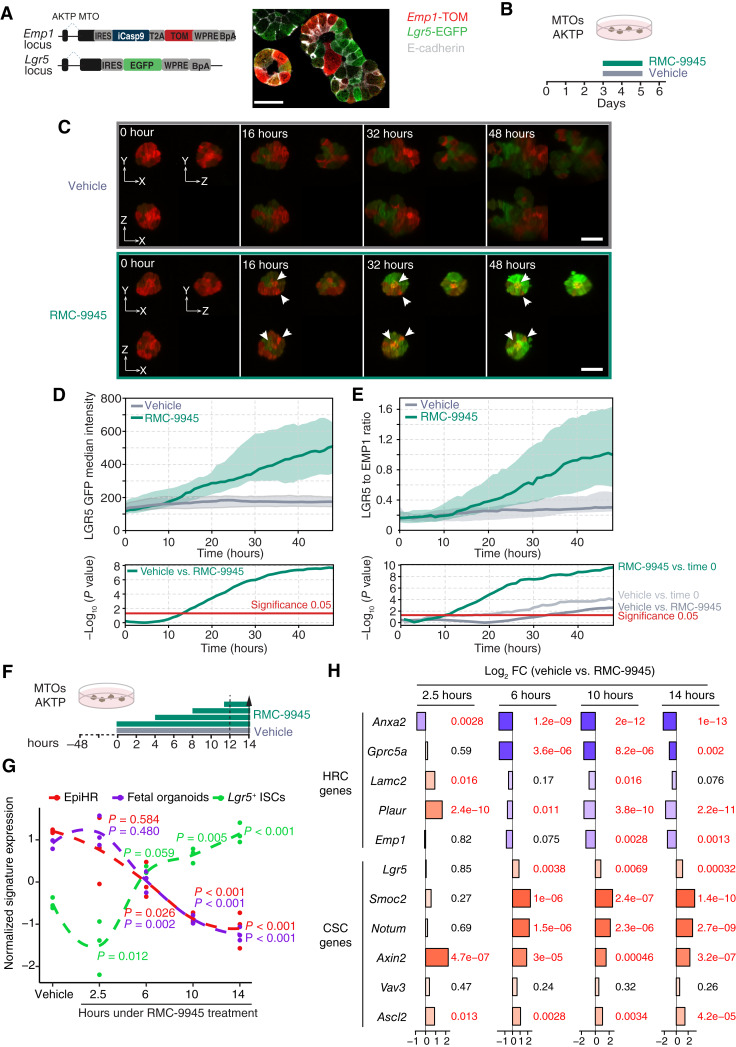
Dynamics of cell state conversion by KRAS inhibition. **A, ***Emp1*-iCasp9-tdTomato and *Lgr5*-EGFP alleles introduced in AKTP MTOs. Confocal imaging of TOM, EGFP, and E-cadherin immunostaining in CRISPR knock-in MTOs. Single z-plane. Scale bar, 30 µm. **B,** Schematic of *in vitro* AKTP MTOs treated with vehicle or RMC-9945 for 48 hours, with media refresh at 12 hours, during live dOPM imaging started at day 3. **C,** Representative 3D orthogonal planes from vehicle-treated (top) and RMC-9945–treated (bottom) organoids. Images show *Lgr5*-EGFP (green)- and *Emp1*-TOM (red)-expressing organoids at 16-hour time intervals over a 48-hour period. Arrows pointing at TOM-expressing cells becoming GFP-high. Scale bar, 50 µm. **D,** Graph showing the median and IQR of the median fluorescence intensity of *Lgr5*-EGFP in vehicle- and RMC-9945–treated AKTP MTOs over a 48-hour period. Vehicle *n* = 20, RMC-9945 *n* = 40. **E,** Graph showing the median and IQR of EGFP/TOM median intensity ratio over a period of 48 hours, measured at 1-hour time intervals upon vehicle and RMC-9945 treatment. EGFP/TOM pixel ratio was normalized at organoid level at time 0. Vehicle *n* = 20, RMC-9945 *n* = 40. Bottom in **D** and **E** show –log10 *P* value derived from Welch *t* test (**D**) and Welch *t* test followed by Wilcoxon signed-rank test (**E**). **F,** Experimental setup of Slam-seq experiments. Dashed line indicates MTO harvesting. **G,** Z-scored and smoothed normalized gene expression of the EpiHR (Cañellas-Socias et al; ref. 13), fetal [Mustata and colleagues (28)], and *Lgr5*^+^ ISCs [Muñoz and colleagues (29)] gene signatures across different time points. *P* values are derived from Dunnett tests. **H,** Barplots showing the Log_2_ fold change (FC) of nascent RNAs upon RMC-9945 treatment at different time points, compared with the vehicle for *Lgr5*^+^ ISC and EpiHR. Wald test–derived significant *P* values are shown in red.

It is formally possible that rather than a plastic cell state conversion, KRAS inhibition results in the specific death of *Emp1*^+^ HRCs, resulting in metastases or organoids enriched in *Lgr5*^+^ CSC cells. To distinguish between plasticity and cell state depletion, we imaged AKTP MTOs bearing *Lgr5*-EGFP and *Emp1*-TdTomato reporters (Fig. 4A–E; ref. 13). For this experiment, we used dual-view oblique plane microscopy (dOPM) that enables 3D live imaging of organoids for several days with low photobleaching and toxicity (40). Figure 4C displays a representative AKTP MTO over 48 hours of either vehicle or RAS(ON) G12D-selective inhibitor RMC-9945 treatment, and the full movie is included as supplementary information (Supplementary Movie S1). At the initial time point, MTOs were formed by a mixture of *Emp1*-TdTomato-bright and *Lgr5*-EGFP-dim and -negative cells (Fig. 4C). This is in accordance with previous observations that mechanical dissociation of organoids downregulates *Lgr5* levels and upregulates the fetal state (21). Under vehicle treatment, a population of *Lgr5*^+^ cells emerged over time, which is consistent with a mechanism of intrinsic plasticity that generates *Lgr5*^+^ CSCs from HRCs as previously reported (21). However, we observed that in RMC-9945–treated MTOs, both *Emp1*-TdTomato-bright and *Lgr5*-EGFP-dim cells underwent a progressive conversion toward an *Lgr5*-EGFP-bright state (Fig. 4C, arrows; Supplementary Movie S1). Measurement of the *Lgr5*-EGFP reporter intensity (Fig. 4D) and the quantification of proportion of *Lgr5*-EGFP and *Emp1*-TdTomato expression (Fig. 4E) showed consistent upregulation of the *Lgr5*-EGFP reporter and a progressive *Emp1*-TdTomato+ to *Lgr5*-EGFP-bright plastic reversion upon KRAS^G12D^ inhibition. OPM imaging for extended time periods (day 9) confirmed that *Lgr5*-EGFP-bright organoids expand under RMC-9945 treatment (Supplementary Fig. S10A and S10B).

We reasoned that changes in cell states tracked by this method may be only evident after sufficient EGFP or TdTomato is accumulated. To obtain more accurate insight into the timing of cell state reversion, we performed Slam-seq [thiol(SH)-linked alkylation for the metabolic sequencing of RNA], a method for direct quantification of newly synthesized messenger mRNAs (Fig. 4F–H; ref. 41). Longitudinal Slam-seq detected increased transcription of the global *Lgr5*^*+*^ ISC signature and silencing of EpiHR and Fetal organoid signatures 6 hours after RMC-9945 supplementation (Fig. 4G). However, some key marker genes of CSCs, such as *Axin2* and *Ascl2*, as well as of the HRC states, such as *Anxa2*, were modified as early as 2.5 hours after RMC-9945 addition (Fig. 4H). Overall, these data reveal the occurrence of rapid transcriptional remodeling that facilitates immediate adaptation to KRAS^G12D^ inhibition.

### Changes in Chromatin Organization and TF Occupancy Triggered by the RAS(ON) G12D-Selective Inhibitor RMC-9945

**Figure 5.**
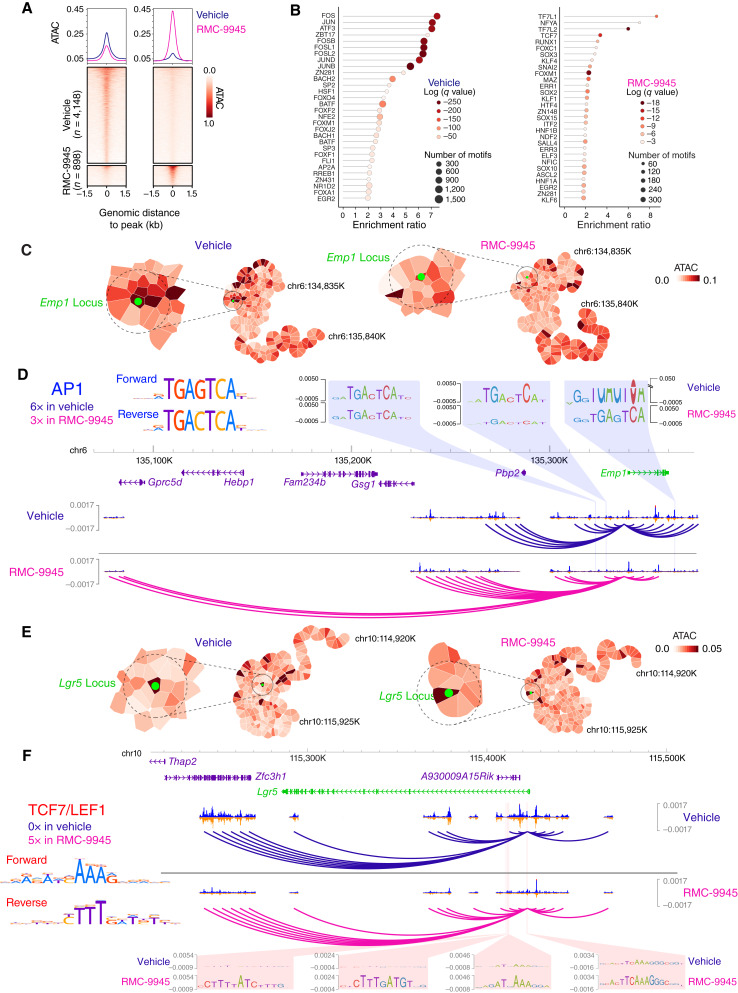
Analysis of chromatin regulation by RMC-9945. **A,** Heatmaps and ATAC profile plots of differential peaks for vehicle-treated (*n* = 4,148) and RMC-9945–treated (*n* = 898) AKTP MTOs for 48 hours. **B,** Motif analysis on differential peaks reveals enrichment in members of the JUN/FOS family of TFs for vehicle (left) and LEF/TCF7 family of TFs for RMC-9945 (right). Top 30 significant most enriched motifs are shown. **C,** Representation (Gaudí plot) for ATAC-seq signal rendered for both the vehicle (left) and RMC-9945 (right), centered at chr6:134,835,000-135,835,000. *Emp1* locus highlighted (green dot). All genomic bins within the dashed circle are taken into consideration for the motif search analysis. **D,** ChromBPNet contribution tracks for vehicle and RMC-9945 highlighting three (of six) AP1 motifs open in vehicle and closed in RMC-9945. Arcs represent the interaction between the TSS containing 5 kbp bin and those in spatial proximity in the Gaudí plot (dashed circle in **C**). **E,** Gaudí plot for ATAC-seq signal rendered for *Lgr5* locus, centered at chr10:114,920,000-115,925,000. Same representation as in **C**. **F,** ChromBPNet contribution and interaction tracks for vehicle and RMC-9945 highlighting four (of five) TCF7/LEF1 TF-binding sites open in RMC-9945.

To characterize how oncogenic KRAS regulates tumor cell plasticity, we combined Assay for Transposase-Accessible Chromatin using sequencing (ATAC-seq) with Hi-C in APTP MTOs (Fig. 5; Supplementary Fig. S11 and S12; ref. 42). At genome-wide level, chromatin accessibility analysis by ATAC-seq identified 4,148 genomic regions that decreased their accessibility after RAS(ON) G12D-selective inhibitor RMC-9945 treatment (Fig. 5A). Inference of TF binding in these regions based on the algorithm MEME (43) showed highly significant enrichment in members of FOS (FOS, FOSB, FOSL1, and FOSL2) and JUN (JUN, JUNB, and JUND) family of TFs (Fig. 5B). FOS and JUN heterodimerize to bind AP1 sequences on regulatory DNA regions and have been previously shown to mediate the transcriptional outputs of KRAS mutations (44, 45). Conversely, there were 898 chromatin regions that became more accessible upon KRAS^G12D^ inhibition (Fig. 5A), showing enrichment in binding sites for the LEF/TCF family of TFs (TCF7, TCF7L1/TF7L1, and TCF7L2/TF7L2; Fig. 5B), which are nuclear effectors of canonical WNT signaling.

To obtain more detailed insight on how these TFs participate in KRAS-mediated plasticity, we analyzed the regulation of the *Emp1* and *Lgr5* genes by integrating ATAC-seq and Hi-C data (Fig. 5C–F; Supplementary Fig. S11A and S11B). The analysis allowed us to assess the spatial correlation of chromatin accessibility signal at 5-kb resolution in the 3D chromatin space using a 2D representation of the Hi-C map based on the Kamada–Kawai graph layout (Supplementary Fig. S11). The graphs were next used to render the ATAC-seq signal in space (Fig. 5C and E). Although the overall topology of the genomic region containing the *Emp1* locus was not significantly altered upon KRAS^G12D^ inhibition (Supplementary Fig. S11A), there was a decrease in openness around the transcription start site (TSS; green dot in Fig. 5C). Our analysis also revealed a rearrangement of the *Emp1* spatial neighborhood upon RAS(ON) G12D-selective inhibitor RMC-9945 treatment, resulting in increased number of interactions with proximal and distal genomic regions with decreased accessibility according to the ATAC-seq signal (dashed circle in Fig. 5C, arcs track in Fig. 5D). To assess whether these changes correlated with differential TF occupancy, we next used ChromBPNet, a convolutional neural network that assesses the nucleotide-resolution contribution to chromatin openness measured by ATAC-seq described by Pampari and colleagues (bioRxiv 2024.12.25.630221). This analysis allowed us to identify TF binding sites in open chromatin regions spatially close to the *Emp1* TSS. For example, there were six binding motifs for AP1 in open chromatin regions in vehicle and three upon KRAS^G12D^ inhibition despite the increased interactions observed under RMC-9945 treatment conditions (Fig. 5D). Using CUT&RUN, we confirmed reduced JUNB binding at several predicted AP1 sites following KRAS^G12D^ inhibition (examples in Supplementary Fig. S12A and S12B). These sites also exhibited decreased H3K27ac marks. A similar pattern was observed when analyzing EpiHR signature genes globally for JUNB binding and H3K27ac levels (Supplementary Fig. S12C).

For the *Lgr5* locus, we did not observe large topologic alterations in its genomic region (Supplementary Fig. S11B), with local and distal interactions of the *Lgr5* locus persisting unaltered upon RMC-9945 treatment (arcs track in Fig. 5F). Integration of ATAC-seq signal into Hi-C showed that the *Lgr5* locus remained in an overall open configuration (Fig. 5E, dashed circle). Yet, the *Lgr5* TSS and its interacting regions clearly increased their accessibility (Fig. 5E, dashed circle). Analysis of the TF footprints in the neighborhood regions identified five *bona fide* TCF/LEF binding sites, four of them located within intron 1 of *Lgr*5 (Fig. 5F), that become occupied in RMC-9945–treated MTOs.

Collectively, the analysis of the regulation of *Emp1* and *Lgr5*, the two key marker genes for HRCs and CSCs, illustrates a plausible mechanism underlying KRAS^G12D^–mediated transcriptional plasticity. This mechanism seems to involve dynamic shifts in TF occupancy—prominently between AP1 and TCF/LEF complexes—requiring limited chromatin rearrangements.

### RMC-9945 Increases β-Catenin/TCF Transcriptional Activity

**Figure 6.**
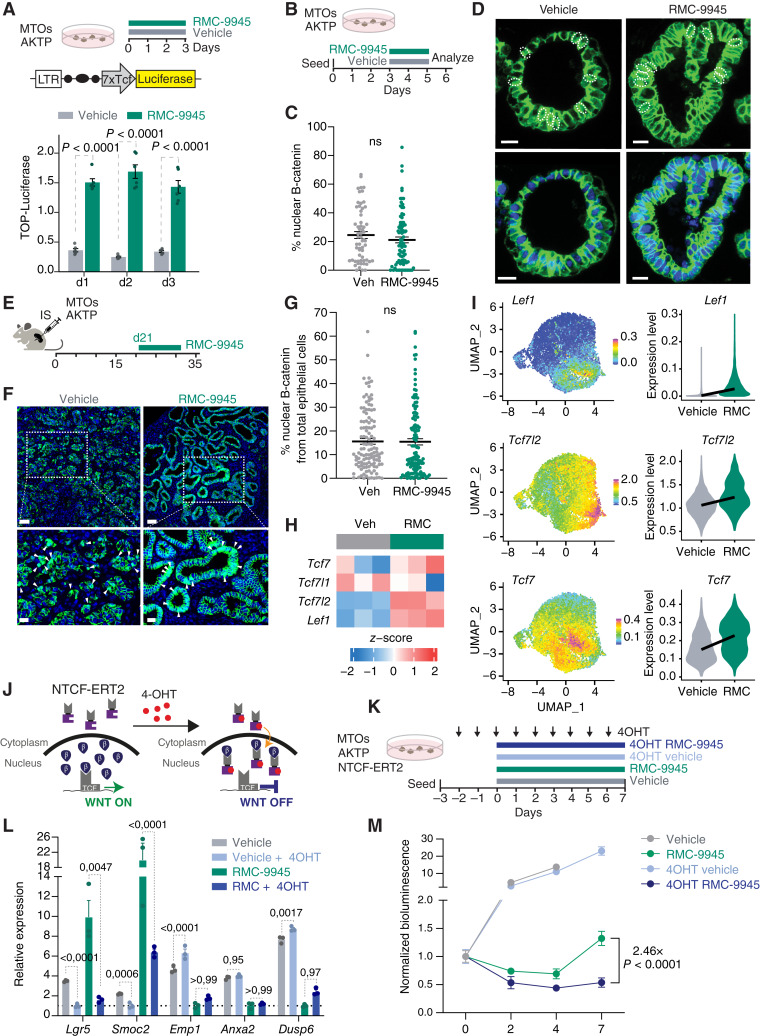
RMC-9945 increases β-catenin/TCF transcriptional activity. **A,** Schematic representation of seven TCF optimal promoter (TOP) luciferase reporter lentiviral vector introduced into AKTP MTOs. TOP-Luciferase quantification of AKTP TOP-Luciferase MTOs, normalized to CellTiter-Glo parallel wells treated with vehicle or RMC-9945 for 1, 2, and 3 days (*n* = 6). Linear mixed-effect model followed by EMMs with pairwise comparisons. **B,** Schematic of *in vitro* AKTP MTOs treated with vehicle or RMC-9945 for 48 hours, with media refresh at 12 hours. **C,** Immunofluorescence image quantification of the percentage of nuclear (4′,6-Diamidino-2-phenylindole, DAPI-positive) β-catenin–positive cells within AKTP MTO vehicle-treated or RMC-9945–treated organoids. **D,** Representative images of the quantification displayed in **C**. Dashed circles illustrate nuclear β-catenin–positive cells. Scale bars: 20 µm. *P* value is derived from unpaired *t* test. **E,** Experimental setup of RMC-9945 treatment of liver metastases. Treatment started at day 21 after inoculation of liver metastases implanted in the spleen of C57BL/6J mice and continued for 11 days. **D** and **F,** Immunofluorescence representative images (**F**), and quantification (**G**) of the percentage of nuclear (DAPI-positive) β-catenin–positive cells within liver metastasis derived from AKTP MTOs, treated with vehicle or RMC-9945. Scale bar, 100 µm top, 20 µm bottom. White arrows highlight examples of nuclear β-catenin–positive cells. *P* value is derived from unpaired *t* test. **H,** Heatmap showing expression of TFs in AKTP MTO organoids treated with vehicle or RMC-9945. **I,** Violin plots showing single-cell expression levels of WNT-related TFs in liver metastases treated with vehicle or RMC-9945. **J,** Schematic representation of the nTCF4-ERT2 construct which translocates into the nucleus and conditionally induces a WNT-OFF state upon treatment with 4-OH-tamoxifen. **K,** Schematic representation of the experiments displayed in **L** and **M,** consisting on nTCF4-ERT2 infected AKTP MTOs treated with 4-OH-tamoxifen in the corresponding conditions since 1 day after being seeded (day -2) and with RMC-9945 or vehicle for 7 days. **L,** Relative mRNA expression of indicated marker genes in vehicle-treated and RMC-9945–treated (with or without 4OHT) AKTP MTOs for 48 hours. Mean ± SEM. *P* values are derived from ANOVA followed by Šídák multiple comparisons test. **M,** AKTP MTO organoid growth (normalized bioluminescence) treated with vehicle, RMC-9945, 4-OH-tamoxifen + vehicle, or 4-OH-tamoxifen + RMC-9945. 4-OH-tamoxifen conditions are normalized to the mean luminescence of 4-OH-tamoxifen treatment at experimental day 0 and non–4-OH-tamoxifen–treated conditions are normalized to the mean luminescence of non–4-OH-tamoxifen treatment experimental day 0. Mean ± SEM; *P* values are derived from ANOVA followed by Šídák multiple comparisons test.

To test the possibility that oncogenic KRAS suppresses the activity of the β-catenin/TCF transcriptional complex, we transfected AKTP MTOs with the TOP reporter vector, which contains multimerized optimal TCF-binding sites driving luciferase expression (46). Treatment with RMC-9945 led to a fivefold increase in β-catenin/TCF activity in AKTP MTOs (Fig. 6A). However, quantification of β-catenin nuclear staining in MTOs and liver metastases showed no differences between vehicle-treated and RMC-9945–treated samples, suggesting that KRAS^G12D^ activity did not promote β-catenin nuclear translocation *per se* (Fig. 6B–G). Instead, RMC-9945 upregulated the mRNA levels of three key nuclear β-catenin partners—the TFs TCF7, LEF1, and TCF7L2—in both AKTP MTOs and liver metastases (Fig. 6H and I).

To directly test the dependency of RMC-9945–induced cell states on β-catenin/TCF transcriptional activity, we generated AKTP MTOs that express a dominant negative TCF factor consisting of the β-catenin binding domain of TCF7L2 fused to the hormone-binding domain of the estrogen receptor (nTCF4-ERT2; Fig. 6J–M). Media supplementation with 4-OH-tamoxifen activates the nTCF4-ERT2 construct which competes for β-catenin binding and prevents β-catenin/TCF-mediated transcription (Fig. 6J; refs. 47, 48). In AKTP MTOs, activation of the nTCF4-ERT2 decreased twofold to threefold the expression of the CSC markers *Lgr5* and *Smoc2* and, importantly, blocked the upregulation of these genes by RMC-9945 to a large extent (Fig. 6L). In contrast, the expression of HRC marker genes remained unmodified (Fig. 6L). These results further confirmed that RMC-9945–mediated induction of CSC program is driven by increased β-catenin/TCF-driven transcription. Of note, MTOs kept expanding in the presence of 4-OH-tamoxifen, implying that they withstand the reduction in β-catenin/TCF activity (Fig. 6M; Supplementary Fig. S13A). In contrast, induction of the nTCF4-ERT2 prevented outgrowth under RMC-9945 treatment (Fig. 6M). Indeed, inspection of MTO cultures showed that most organoids in the combinatorial treatment were necrotic (Supplementary Fig. S13B and S13C, arrows). These findings support that the activity of the β-catenin/TCF transcriptional complex is necessary to resist the effects of blocking oncogenic KRAS activity.

### Dependency on *Lgr5*^+^ CSCs in RMC-9945 Treated Metastasis

**Figure 7.**
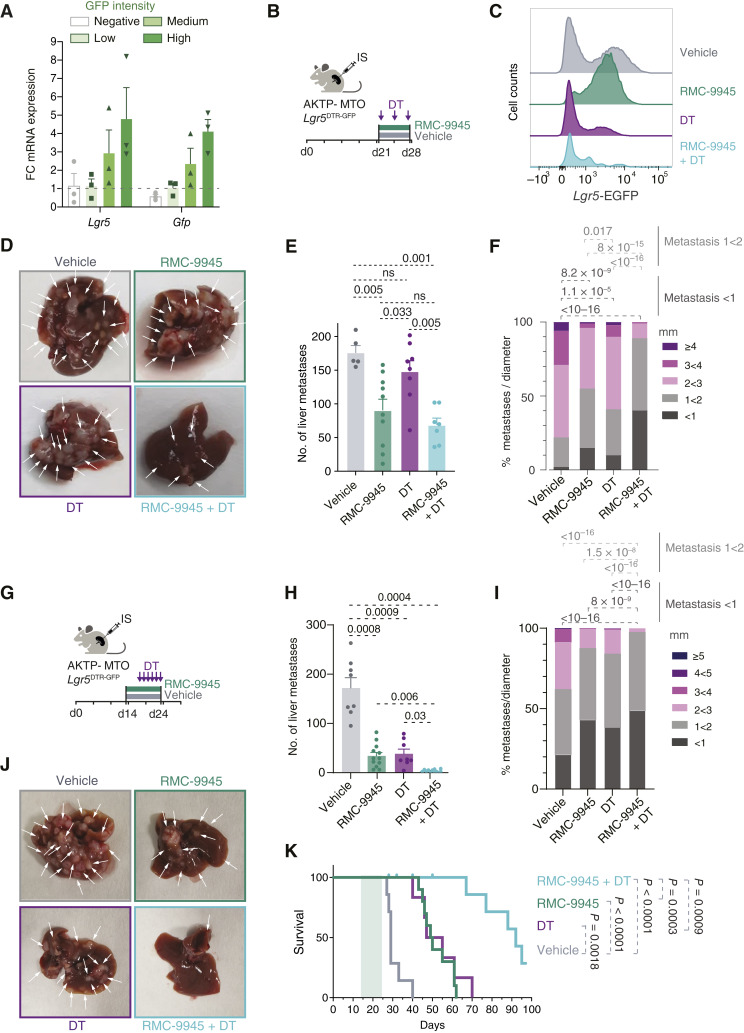
Dependency on *Lgr5*^+^ CSCs in RMC-9945–treated metastases. **A,** Relative mRNA expression of *Gfp* and *Lgr5* genes in sorted GFP-high, -medium, -low, and -negative population from AKTP MTO-derived liver metastases. Mean ± SEM. **B,** Schematics of *in vivo* RMC-9945 treatment (7 days, starting at day 21 after implantation), Diphtheria toxin (DT) treatment (one injection any other day), or combination of RMC-9945 and DT, of liver metastases implanted in the spleen of C57BL/6J mice. **C,** Representative histograms of *Lgr5*-EGFP expression of the EPCAM^+^/CD45^−^ population in mice treated with vehicle, RMC-9945, DT, and RMC-9945 + DT obtained by flow cytometry. **D,** Representative pictures of livers after 7 days of treatment with vehicle, RMC-9945, DT, and RMC-9945 + DT. Arrows pointing at liver metastases. **E** and **F,** Quantifications of total number of metastases per mouse in each condition described (**E**) and of the proportion of metastases per size (higher diameter; **F**). Vehicle *n* = 5 mice, RMC-9945 *n* = 10, DT *n* = 8, and RMC-9945 + DT = 7. Mean ± SEM. For **E, ***P* values are derived from ANOVA followed by Tukey tests. For **F**, *P* values are derived from the β-binomial regression model. **G,** Schematics of *in vivo* RMC-9945 treatment (10 days, starting at day 15 after implantation), DT treatment (one injection every day), or combination of RMC-9945 and DT, of liver metastases implanted in the spleen of C57BL/6J mice. **H** and **I,** Quantifications of total number of metastases per mouse in each condition described (**H**) and of the proportion of metastases per size (higher diameter; **I**). Vehicle *n* = 8 mice, RMC-9945 *n* = 12, DT *n* = 8, and RMC-9945 + DT = 12. Mean ± SEM. For **H, ***P* values are derived from ANOVA followed by Dunnett tests. For **I**, *P* values are derived from the β-binomial regression model. **J,** Representative pictures of livers after 10 days of treatment with vehicle, RMC-9945, DT, and RMC-9945 + DT. Arrows pointing at liver metastases. **K,** Kaplan–Meier survival plot of mice treated with the indicated treatments. *P* values are derived from Gehan–Breslow–Wilcoxon test.

Our findings imply that the plastic HRC-to-CSC reversion observed upon RMC-9945 treatment represents a mechanism of resistance to mutant KRAS inhibition in mCRC. It follows that RMC-9945 may likely synergize with therapies targeting the *Lgr5*^+^ CSC state. To test this hypothesis, we made use of AKTP MTOs engineered with an EGFP plus a diphtheria toxin receptor (DTR) bicistronic cassette inserted into the *Lgr5* locus using CRISPR (Supplementary Fig. S14A; ref. 13). In this model, *Lgr5*^+^ CSCs express both EGFP and DTR, and therefore, this population is susceptible to killing by addition of DT. *In vitro*, RMC-9945 synergized with DT treatment to eliminate most MTOs (Supplementary Fig. S14B and S14C). *Lgr5*-DTR-EGFP MTOs generated metastases that include EGFP-labeled *Lgr5*^+^ CSCs, as confirmed by RT-qPCR analysis from sorted tumor cells expressing high, medium, and low GFP levels (Fig. 7A). In these experiments, treatment was initiated on day 21, when mice harbored more than 100 large liver metastases. Flow cytometry analysis of dissociated metastases showed that *Lgr5*-EGFP^+^ tumor cell population was almost completely depleted after DT inoculation (Fig. 7B and C). At experimental endpoints, RMC-9945 monotherapy reduced by twofold the number of liver nodules, yet an overt macrometastatic disease was still present (Fig. 7D–F; Supplementary Fig. S14D). As predicted from our previous results, RMC-9945 treatment homogenized metastatic cells to a *Lgr5*-EGFP^+^ cell state (Fig. 7C). The combination of RMC-9945 plus DT-mediated ablation of *Lgr5*^+^ CSCs exerted a robust therapeutic effect, with threefold reduction in metastasis size (Fig. 7E and F; Supplementary Fig. S14D–S14F). This effect extended mouse survival after compound withdrawal; however, in the absence of treatment, residual metastatic nodules expanded rapidly, and animals succumbed to metastatic disease (Supplementary Fig. S14E).

We also tested the above combinatorial strategy by initiating treatment at day 14 after inoculation. In this earlier setting, RMC-9945 arrested metastatic outgrowth (Fig. 1B and C). However, as shown previously, metastases were not eradicated but persisted as nodules enriched in *Lgr5*^+^ drug-tolerant persister cells (Fig. 1O–Q). DT-mediated ablation of *Lgr5*^+^ cells also led to a significant reduction in metastatic burden (Fig. 7G–J). The combination of RMC-9945 and DT reduced the metastatic load 10- to 11-fold compared with either monotherapy (Fig. 7H–J). To evaluate the curative potential of this treatment, we withdrew administration of RMC-9945 and DT. Mice receiving monotherapy died approximately 40 days after RMC-9945 or DT treatment cessation, whereas those treated with the RMC-9945 plus DT combination survived for markedly extended periods (Fig. 7K).

## Discussion

Over the past few years, it has become increasingly clear that transcriptional plasticity is pervasive in colorectal cancer (10, 24, 25). A large body of evidence supports a key role for tumor cells co-opting a CSC state driven by WNT pathway–activating mutations in most colorectal cancers (11). However, recent findings have shown that a subset of cells can abandon the WNT-activated CSC state and adopt a distinct transcriptional program that has been experimentally linked to metastasis (13, 18, 20, 21) and chemotherapy resistance (15–17, 23). The determinants of such plastic transitions are currently under intense investigation. Previous studies have demonstrated that CSC-to-HRC conversion can be triggered by microenvironmental signals emanating from the cancer-associated fibroblasts (CAF) niche (13, 23), chemotherapy (16, 17, 23), or mechanical cues (14). In this study, we reveal a key role for activating *KRAS* mutations in mediating this process. The observations described herein have several implications for understanding the role of cell state plasticity in colorectal cancer progression and therapy resistance.

First, we show that activating *KRAS* mutations lower β-catenin/TCF transcriptional activity in mCRC cells and promote the expression of genes encoding the HRC state. Whereas we favor a definition of HRCs based on the upregulation of a poor prognosis gene program (EpiHR; ref. 13), they overlap with the so-called oncofetal and regenerative stem cell states described by several laboratories in recent publications (15, 18, 19). All these malignant cell states are marked by high *Emp1* levels and downregulation of WNT/CSC core genes such as *Lgr5* (15, 18, 19). We show that pharmacologic suppression of mutant KRAS restores WNT/CSC dependency in colorectal cancer. Through scRNA-seq analysis and genetic ablation experiments, we demonstrate that the acquisition of an *Lgr5*^+^ CSC state enables metastases to withstand the deleterious effects of KRAS^G12D^ inhibition. These preclinical data suggest that metastatic disease in patients with colorectal cancer may likely resist KRAS-based therapies through a similar mechanism. Fittingly, a recent analysis by Villarreal and colleagues (bioRxiv 2025.01.22.634215) of colorectal cancer patient samples enrolled in a clinical trial of combined BRAF and MEK inhibition linked drug resistance to the plastic acquisition of a stem cell–like transcriptional state. This cell state closely resembles the *Lgr5*^+^ CSC program described in our study, reinforcing its relevance in therapeutic resistance. We propose that combining KRAS^G12D^ inhibitors with therapies targeting CSCs may produce potent therapeutic responses in patients with mCRC. In this regard, a recent study showed that KRAS^G12C^ inhibitors synergize with EZH2 inhibitors to drive differentiation and apoptosis in colorectal cancer cell lines (49).

Second, at the genome-wide level, we observed a rapid change of the metastatic transcriptional program following RMC-9945 treatment. *Emp1* and *Lgr5*, the two key marker genes for HRCs and CSCs, become activated and suppressed, respectively, without apparent large-scale chromatin remodeling. However, the integration of Hi-C and ATAC-seq analysis demonstrates that despite these modest chromatin changes, gene regulatory networks based on TF occupancy respond to the RMC-9945 treatment. Indeed, HRC gene regulatory regions are enriched for binding sites of the FOS and JUN (AP1) family of TFs, which are downstream effectors of MAPK signaling (44, 45). This observation is consistent with recent studies linking the oncofetal state to AP1 TFs (19). In contrast, the ISC-like program that emerges upon KRAS inhibition is enriched in binding motifs for LEF/TCF TFs, which interact with β-catenin to drive WNT-driven transcription downstream of pathway-activating mutations. These findings suggest that colorectal cancer cells adopt a transcriptional state that reflects a balance between KRAS/AP1 and WNT/TCF signaling. As both opposing pathways are constitutively activated in colorectal cancer as a result of genetic alterations, additional extrinsic or intrinsic cues may further influence the plastic transition from CSCs to HRCs. Although not investigated herein, such extrinsic cues, including interactions with CAFs, inflammatory signals, and hypoxia, have been previously associated with CSC-to-HRC plasticity (13, 15, 16, 18, 19, 23, 50). It is possible that mutations activating the RTK/RAS signaling pathway enable tumor cells to co-opt these transcriptional programs independently, which are then further reinforced or modulated by TME-derived signals *in vivo*. Dissecting the interplay between oncogenic signaling and TME-derived cues in regulating CSC-to-HRC/oncofetal transitions will be essential to fully understand plasticity-driven drug resistance and represents a key area for future investigation.

Third, long-standing evidence supports that transcriptional heterogeneity in colorectal cancer reflects the expression of stem cell and differentiation gene programs reminiscent of the healthy mucosa (11). However, HRCs are neither differentiated nor stem-like. Rather, a KRAS^G12D^–dependent transcriptional mechanism lowers the expression of multiple TFs that define the intestinal epithelium in this population. We show that KRAS-activated HRCs express several genes representative of PDAC. For example, EpiHR poor prognosis–associated genes such as *MSLN* (51), *PLAUR* (52), and *LAMC2* (53) play important roles in PDAC progression. These parallels may simply reflect shared oncogenic KRAS-driven programs in both tumor types. In addition, we also report *de novo* expression of keratins and keratinization mediators in a subset of HRCs, suggesting that certain tumor cells may co-opt a squamous gene program. Overall, these findings align with recent scRNA-seq analyses of patients with mCRC, which revealed abundant noncanonical (nonintestinal) cell states in metastases as a result of increased plasticity (18). Activity of the AP1 complex has also been shown to promote the acquisition of an esophageal cancer-like program in colorectal cancer organoids (19). A recent study also showed that mutations in the chromatin remodeler ATRX lead to loss of intestinal lineage genes and expression of a squamous program (38). Therefore, we speculate that *KRAS* mutations contribute to the lineage infidelity observed in metastases, whereas KRAS inhibition reestablishes an intestinal epithelium-like state driven by *Lgr5*^+^ stem cell–like tumor cells.

Whereas the *EMP1*^+^ HRC to *LGR5*^+^ CSC transition enables tolerance to KRAS^G12D^ inhibition, it does not, by itself, guarantee proliferative escape. We found that the fate and behavior of the reprogrammed *Lgr5*^+^ cells differ markedly depending on disease stage. When RMC-9945 is administered early (day 15), *Lgr5*^+^ cells adopt a drug-tolerant phenotype but fail to drive metastasis outgrowth. In contrast, treatment at later stages (day 21 or 25) results in *Lgr5*^+^ cells that remain proliferative under KRAS inhibition, sustaining disease progression. We speculate that this differential behavior may be governed by changes in TME over metastatic progression: early metastases may lack proliferative cues present in more established lesions, promoting stasis rather than expansion of the *Lgr5*^+^ population. More broadly, our results suggest that the biological consequences of KRAS^G12D^ inhibition are highly context-dependent, varying not only by timing but also by tumor model. Whereas some colorectal cancer models exhibit transient cytostasis followed by expansion of *Lgr5*^+^ cells, others display immediate progression under treatment. Furthermore, during prolonged in vitro treatment, we observed acquisition of an initial *Lgr5*^+^ CSC quiescent state followed—during regrowth—by progressive decline of the *Lgr5*^+^ program and reacquisition of the *Emp1*^+^ HRC state, consistent with reactivation of MAPK signaling. Nevertheless, all these distinct behaviors converge on a shared plastic transition to a WNT-driven *Lgr5*^+^ CSC state that enables tumor cells to resist the deleterious effects of inhibiting oncogenic KRAS signaling. Future work should define therapeutic strategies to target the LGR5^+^ CSC state as a means to potentiate KRAS^G12D^ inhibition in mCRC.

Recent preclinical studies have investigated resistance to KRAS inhibitors in lung adenocarcinoma (LUAD), PDAC, and NSCLC models. In LUAD, KRAS inhibition promotes the transition of tumor cells to an alveolar type 1–like state, which is resistant to therapy. These cells can regenerate the disease after treatment cessation (54). In PDAC, basal cells are sensitive to KRAS^G12D^ inhibition, yet tumor cells expressing a classic PDAC program are resistant to therapy and serve as a reservoir for disease relapse (55, 56). In *KRAS*-mutant NSCLC, a mucinous transcriptional state tolerates treatment with a RAS(ON) multiselective inhibitor (57). Therefore, mirroring our observations in mCRC models, PDAC, LUAD, and NSCLC harbor tumor cells in transcriptional states with different degrees of oncogenic KRAS dependency. Beyond advancing our understanding of resistance to KRAS-targeting therapies, these findings highlight the importance of transcriptional context in determining the effect of oncogenic *KRAS* mutations across tumor types and positions tumor cell plasticity at the core of therapeutic resistance.

## Methods

### Ethics and Animal Maintenance

All experiments with mouse models were approved by the Animal Care and Use Committee of Barcelona Science Park (CEEA-PCB) and the Catalan government. Mice were maintained in a specific pathogen-free facility with a 12-hour light–dark cycle and given *ad libitum* access to standard diet and water. All of the mice were closely monitored by the authors, facility technicians, and an external veterinary scientist responsible for animal welfare.

### Mouse Splenic Injections

For all injections, genetic models or C57BL/6J mice (RRID: IMSR_JAX:000664, purchased from Janvier Labs at 6 weeks of age) were injected at 7 to 8 weeks. Sex was matched with the origin of the tumor. Intrasplenic injections were used for liver colonization by the introduction of dissociated organoids (small 3–4 cell clusters) into the portal circulation. MTOs were cultured in standard six-well plates for 3 days and trypsinized as described in “MTO Culture”. Cells were counted and suspended in Hank’s Balanced Salt Solution (HBSS) for injection, using 1  ×  10^5^ cells in 70 μL/mouse. Cell viability was counted using Trypan Blue 0.4% (Gibco, 15250061) and a TC20 automated cell counter (Bio-Rad). Intrasplenic injections were performed as previously described (27). After injection, all mice received analgesia (buprenorphine). Mice were euthanized at 5 to 6 weeks according to the treatment regimen, and visible liver metastases were counted.

### Metastasis Tracking Using Bioluminescence Imaging

For *in vivo* monitoring of tumor growth, MTOs were previously engineered to constitutively express the luciferase enzyme (27) and were tracked with *in vivo* bioluminescence using an IVIS-Spectrum (Perkin-Elmer) imager and Living Image software (v4.5.2, Perkin-Elmer). Animals were anesthetized with 2.5% isoflurane and received a retro orbital injection with 50 μL D-luciferin at 15 mg/mL (Resem BV). Mice were shaved before measurement, when necessary, using electrical trimmers. For quantification, images (typically in the 0.5–60 seconds exposure range, bin 4–16) were quantified using the total flux (photons second^−1^) of a region of interest (ROI) spanning the lower thorax and upper abdomen. Data were processed and visualized with GraphPad Prism (v10.2.3, RRID: SCR_002798).

### *In Vivo* Treatments

For *in vivo* treatment, RMC-9945 was dissolved in 10% DMSO, 20% polyethylene glycol 400, 10% Solutol HS15, and 60% water. *In vivo* formulations were stored at 4°C for 1 week. Mice were treated daily with RMC-9945 or vehicle at a 100 mg/kg by oral gavage. For DTR-inducible ablation, mice were treated with 25 μg/kg of DT (Sigma-Aldrich, 322326) every other day for 2 weeks or every day for 6 days.

### Immunohistochemistry/Immunofluorescence

Livers extracted from mice were fixed in a 10% formalin solution (Sigma, HT501128) or 4% paraformaldehyde in PBS (ChemCruz, sc-281692) O/N at room temperature (RT) and washed with PBS. Fixed tissues were embedded in paraffin blocks, and 3-μm sections were cut by the histopathology facility at IRB Barcelona. Sections were deparaffinized by serial washes on solutions with a decreasing percentage of alcohol. For antigen recovery, deparaffinized sections were either boiled in a Tris-EDTA solution (Sigma, Tris: T6066, EDTA: E5134) for 20 minutes or autoclaved for 20 minutes at 1 atm of pressure in a citrate buffer at pH 6. Sections were washed using a wash buffer solution from DAKO (K800721), and endogenous peroxidase was blocked using a peroxidase-blocking solution from DAKO (S202386) at RT for 10 minutes. For IHC staining, after incubation with the primary antibody, sections were incubated with a peroxidase-conjugated secondary antibody (horseradish peroxidase) for 30 minutes at RT and revealed using a DAB (3,3′-Diaminobenzidine) solution (DAKO, ref: K346811) at RT. Sections were then washed with distilled water and stained with hematoxylin (Panreac, 251344.1606) for 2 minutes at RT. Finally, stained sections were dehydrated by serial washes with alcohol solutions of increasing percentages and mounted with DPX (Panreac, 255254.1608). For immunofluorescence staining, sections incubated with the primary antibody anti-tdTomato (SICGEN, cat. #AB8181, RRID: AB_2722750), anti–E-cadherin (Abcam, cat. #ab15148, RRID: AB_301693), and non-phospho (active) β-catenin (Ser33/37/Thr41; D13A1) rabbit mAb (Cell Signaling Technology, RRID: AB_11127203) were incubated with secondary antibodies in DAKO diluent (DAKO, K8006) for 1 hour at RT. The following secondary antibodies were used: donkey anti-goat conjugated to Alexa 488/568/647 (Life Technologies–Thermo Fisher Scientific, A11055 RRID: AB_2534102, A11057 RRID: AB_2534104, and A21447 RRID: AB_2535864), donkey anti-rabbit conjugated to Alexa 488/568/647 (Life Technologies–Thermo Fisher Scientific, A21206 RRID: AB_2535792, A10042 RRID: AB_2534017, and A31573 RRID: AB_2536183) and donkey anti-mouse conjugated to Alexa 488/568/647 (Life Technologies–Thermo Fisher Scientific A-21202 RRID: AB_141607, A10037 AB_11180865, and A31571 RRID: AB_162542) at RT. After incubation with the secondary antibody, nuclei were stained with a 400 ng/mL DAPI solution (Sigma, D9542-1MG) in DAKO wash buffer for 10 minutes at RT. Finally, stained sections were mounted using a fluorescent mounting medium (DAKO, S302380-2). Digital scanned brightfield and fluorescent images were acquired with a NanoZoomer-2.0 HT C9600 scanner (Hamamatsu Photonics). All of the images were visualized with the NDP.view 2.0 U123888-01 software (Hamamatsu Photonics) with a γ correction set at 1.8 in the image control panel. AKTP MTO images were taken using a 1 × 81 Olympus ScanR microscope and analyzed with the RT Xcellence Software (v1.2).

### *In Situ* Hybridization – RNAscope

Samples were fixed overnight at 4°C with neutral-buffered formalin (HT501128-4L, Sigma-Aldrich). All samples were embedded in paraffin using routine procedures. Paraffin-embedded tissue sections (2–3 μm in thickness) were air-dried and further dried at 60°C overnight prior to any staining. Ready-to-use reagents from RNAscope 2.5 LS Reagent Kit-RED (322150, RNAScope – ACD Bio-Techne) were loaded onto the Leica Biosystems’ BOND RX Research Advanced Staining System according to the user manual (Doc. No. 322100-USM). FFPE tissue sections were baked and deparaffinized on the instrument, followed by epitope retrieval (using Leica Epitope Retrieval Buffer 2 at 95°C for 15 minutes) and protease treatment (15 minutes at 40°C). Probe hybridization, signal amplification, colorimetric detection, and counterstaining were subsequently performed following the manufacturer’s recommendations. Hybridization was performed with the RNAscope LS 2.5 Probe – Mm-Lgr5 – *Mus musculus* leucine-rich repeat containing G protein–coupled receptor 5 (312178, RNAScope – ACD Bio-Techne). Control probe used was the RNAscope 2.5 LS Probe – Mm-UBC – *Mus Musculus* ubiquitin C (Ubc) as a housekeeping gene (310778, RNAScope – ACD Bio-Techne).

### Histopathologic Quantifications

Scanned immunofluorescence slides were analyzed in QuPath (v.0.5.1) using the positive cell and the subcellular detection with empirical parameters. Several annotation ROIs (tumors) were taken per section. Data were processed and visualized with GraphPad Prism software (v10.2.3; see “Statistics and reproducibility” for more details).

### Flow Cytometry

Macrodissected liver metastases were chopped with scalpels. Subsequent enzymatic digestion was performed with 200 U/mL collagenase IV (Sigma, C5138), 200 mg/mL Dispase II (Sigma, D4693-1G), and 40 μg/mL DNase I (Roche, 10104159001) in HBSS (Gibco, 14175-053) for 60  minutes at 37°C in a shaking water bath. Digested tissue fragments were quenched and filtered through a 70-μm cell strainer. Red blood cells were then lysed using ammonium chloride and subsequently washed with FACS Buffer (PBS with 1% BSA (Roche, 10735086001), 2 mmol/L EDTA (Sigma, E5134-500G), and 0.05% NaN_3_ (Sigma, S2002-25G)). Samples were Fc-blocked for 15 minutes with anti-CD16/32 (93, eBioscience), and cells were stained with APC anti-EPCAM (BioLegend, 118214) and BV-605 anti-CD45 concentrated 1:200 for 30 minutes at 4°C. Dead cells were stained with 1 µg/mL DAPI (Sigma Aldrich, D9542). Flow cytometry analysis and cell separation were performed in a BD FACSAria Fusion Flow Cytometer (Beckton Dickinson). Data were analyzed using FlowJo software (v10, RRID: SCR_008520). Data were processed and visualized with GraphPad Prism software (v10.2.3; see “Statistics and reproducibility” for more details).

### MTO Culture

For MTO passaging, basement membrane extract (BME; Cultrex BME Type 2, 3533-005-02) drops were incubated with TrypLE Express Enzyme (1X), no phenol red (Life Technologies, 12604039) for 20  minutes at 37°C, followed by mechanical disaggregation of organoid fragments (by pipetting) until a single-cell suspension was obtained. TrypLE was quenched with HBSS (Gibco, 14175-053), centrifuged, and resuspended in 70% cold BME/medium in preheated six-well culture plates. MTOs were cultured in medium [comprised of Advanced DMEM/F12 (Gibco, 12634028) supplemented with 10 mmol/L HEPES (Gibco, 11560496), Glutamax (Gibco, 11574466), B-27 without retinoic acid (Gibco, 12587-010), 50  ng/mL recombinant human EGF (Peprotech, AF-100-15), 100  ng/mL recombinant human NOGGIN, and 1 µmol/L galunisertib (LY2157299)]. NOGGIN was produced in-house (27). Cultures were checked bimonthly for *Mycoplasma* contamination.

### *In Vitro* Treatments

For all the experimental procedures described, AKTP MTOs were treated 72 hours after seeding. MTOs were treated with vehicle (DMSO), RMC-9945 (100 nmol/L), or MRTX1133 (HY-134813 MedChem Express, 1 µmol/L) for 48 hours, and treatment was renewed every 24 hours. For DT experiments, one DT administration (100 ng/mL, Sigma-Aldrich, 322,326) was given to AKTP MTOs, and the analysis was performed 24 hours after.

### MTO dnTCF Experiments

AKTP MTOs were infected with a puromycin-selectable lentivirus with a 4-tamoxifen-inducible dominant-negative TCF4 TF, which consisted in the β-catenin–binding domain of TCF4 used to a modified hormone-binding domain of the estrogen receptor (nTCF-ERT2; refs. 47,58). Positively infected organoids were selected and cultured in 66% BME drops and MTO culture medium with 2 µg/mL puromycin. For the experiment, organoids were disaggregated with TriplE and seeded at 100 cells/μL in 66% BME, with MTO culture medium. Wnt blockade was induced in the corresponding conditions by the addition of 1 µmol/L 4-hydroxytamoxifen (Merck H7904) 24 hours after seeding. A total of 100 nmol/L RMC-9945 and vehicle (DMSO) treatments were started 72 hours after cell seeding, and treatment was renewed every 24 hours. Organoid growth was addressed by CellTiter-Glo (described below) at days 0, 3, 5, and 7 after RMC-9955 treatment start. RT-qPCR data were analyzed at day 2.

### MTO TCF-Luc

AKTP MTOs were infected with a puromycin-selectable lentivirus containing a TCF-luciferase reporter, consisting of seven TCF-binding sites preceding the FireFly luciferase cDNA (addgene, 24308). To assess TCF-luciferase activation, cells were seeded at 300 cells/μL at treated either with vehicle or RMC-9945 for 24, 48, or 72 hours. Luciferase was analyzed using the Luciferase Assay System (Promega, E1500), and the luciferase reading was compared with the cell viability measurement using CellTiter-Glo (described below).

### PDOs

The collection of patient data and tissues for the generation and distribution of organoids was performed according to the guidelines of the European Network of Research Ethics Committees, following European, national and local laws, followed by written informed consent from the patients. PDO_C31M has been previously described (59) and was established by the Hub Organoids (HUB). The Biobank Research Ethics Committee of the UMC Utrecht (TCBio) approved the biobanking protocol under which this research was performed (12–093 HUB-Cancer). PDO_131 was derived from a patient with colorectal cancer subjected to surgery and provided by Biobank IdiPAZ (PT23/00028), integrated into the Spanish Biobank Network (www.rebdiobancos.es). Donors signed informed consent. PDOs were cultured in 70% cold BME/medium drops and complete organoid colorectal cancer tumor medium (CTM) containing AdvancedDMEM/F12, Hepes, Glutamax, B27, nicotinamide 10 mmol/L, N-Acetylcysteine 1.25 nmol/L, 50 ng/mL EGF, R-spondin1 1 µg/mL, Noggin 100 ng/mL, SB202190 10 µmol/L, gastrin 10 nmol/L, A83-01 500 nmol/L, prostaglandin E2 2.5 µmol/L and Normocin 100 μg/mL. For RMC-9945 experiments, 7-day grown organoids were disaggregated with TrypLE, counted, and plated in 70% BME at 830 cells/μL density in complete CTM with ROCK inhibitor Y27632 10 µmol/L. After 3 days, media were changed and cells were treated with RMC-9945 100 nmol/L or DMSO during 4 days with a daily media change.

Colorectal cancer PDXO model CTAX91 (*BRAF*^*V600E*^) and CTAX127 (*BRAF*^*V600E*^) were derived from a collection of colorectal cancer PDXs from Héctor G. Palmer’s laboratory and used for *in vitro* experiments. Samples were collected from patients at Vall d’Hebron University Hospital. The studies were conducted in accordance with the Declaration of Helsinki ethical guidelines. Written informed consent was obtained from all patients, and the study was approved by the Research Ethics Committee of the Vall d’Hebron University Hospital, Barcelona, Spain [approval ID: PR(AG)309/2022]. The PDX models used were derived from BRAF treatment–progressed patient liver metastases. A total of 2 × 10^3^ cells were plated in a 5 µL drop of Matrigel (Corning, 356255; cells diluted in basal media and mixed with Matrigel in a proportion 1:4) in a 96-well plate and cultured with HISC media (60) with rock inhibitor Y27632 10 μmol/L and GSK-3 inhibitor CHIR99021 5 μmol/L (Sigma-Aldrich, SML 1046) for 24 hours. The next day, organoids were cultured under EGF-limiting conditions (HISC media without EGF). After overnight, cells were treated with cetuximab (100 µg/mL; MERCK, 658752), encorafenib (1 µmol/L; MedChemExpress, HY-15605), or vehicle in complete HISC media. The treatments were refreshed every 2 to 3 days. Cell viability was determined on days 0, 3, 5, 7, and 10 using the CellTiter-Glo assay (Promega, G7571) following the manufacturer’s protocol.

### Luminescent Cell Viability Assay

To measure cell viability, we used CellTiter-Glo Luminescent Cell Viability Assay (Promega, G7571). After treatment (refer to the “In Vitro Treatments” section), cells were lysed with 100 µL of CellTiter-Glo directly on the plate, and luminescence was measured after 20 minutes of incubation on an orbital shaker (70 rpm, RT). Luminescence was read using the GloMax Navigator software accordingly with the manufacturer’s instructions.

### dOPM Time-Lapse Imaging and Analysis

dOPM is a light sheet–based fluorescence microscopy technique compatible with flat-bottomed multiwell plate formats (40). dOPM imaging was performed with a Nikon 60x/1.2NA water immersion primary objective and a 50x/0.95NA air immersion secondary objective as described in (61) and using a 488- and 561-nm laser for excitation to monitor GFP and dTomato expression, respectively. dOPM images were acquired at ±35° to the focal plane of the primary objective. EiCT-LiG MTOs were allowed to grow for 3 days before dOPM imaging. Twenty control organoids and 40 100 nmol/L RMC-9945–treated organoids were selected for imaging within the range 0 to 200 μm from the coverslip during a prefind procedure using a 20× air lens for brightfield and epifluorescence imaging.

For 3D time-lapse imaging, all organoids were imaged using dOPM once prior to addition of the vehicle or 100 nmol/L RMC-9945 to establish the baseline fluorescence signal. Afterward, imaging was conducted at 1 or 2-hour intervals over a continuous period of 48 hours or for a period of 232 hours with media change every 2 days, under controlled environment conditions, including temperature 37°C, elevated humidity, and 5% CO_2_ (with cage incubator and stage-top incubator with digital CO_2_ mixer, OKOLAB).

For each dOPM view, z-stack planes were acquired every 2 µm and covered a scan range of 150 µm along each view’s optical axis by remote refocusing. The raw camera pixel size for each dOPM view was 0.35 µm over a 512 × 512 pixel field of view. The illumination light sheet used had a calculated full width at half-maximum of 3 μm at the waist in the sample plane at 488 nm. To co-register the dual view information, TetraSpeck Microspheres (0.2 µm diameter fluorescent beads) were mixed with BME with a stock dilution of 20:1. Following a series of affine transformations described in ref. 40, the two dOPM view 3D datasets were fused into a single 3D dataset in lab coordinates with isotropic voxels of size 0.69 µm in each dimension using the Multiview Fusion plugin in ImageJ (RRID: SCR_003070; ref. 62) and saved as tiff z-stacks for each organoid, time, and channel combination.

3D segmentation was performed using Python and the NumPy module (RRID: SCR_00805). The background offset was subtracted as the first step. For quantifying the presence of a fluorescent reporter in each voxel, a manually defined threshold of 78 digital numbers was applied separately to the EGFP and TdTomato channels. This threshold was used for binary classification, globally, across all organoid, channel, and time point combinations. The median intensity was calculated for the respective 3D binary masked areas for each organoid, time, and channel combination.

### Gene Expression Analysis Using RT–qPCR

RNA from *in vitro* MTO, PDO, and PDXO culture was extracted using the PureLink RNA Mini Kit (Life Technologies, 12183025), whereas RNA from 10.000 EPCAM^+^/CD45^−^ sorted cells was extracted using Single Cell RNA Purification Kit (Norgen, 51800) and DNAse-treated using RNase-Free DNase I Kit (Norgen, 25710). cDNA synthesis was performed using the High Capacity cDNA Reverse Transcription Kit (Life Technologies, 4368813) following the manufacturer’s specifications. RT-qPCRs were performed using the PowerUp SYBR Green Master Mix (Applied Biosystems, 15350929) in triplicates and run using the QuantiStudio 6 Flex instrument (Thermo Fisher Scientific). Gene expression levels were normalized using the reference gene *Tbp* for mouse and *B2M* and *HPRT1* for human samples. See Supplementary Table S5 for used probes.

### RNA-seq and Analysis

We used RNA-seq to profile RMC-9945 effects on AKTP MTOs and APK CTOs and profile the effects of the KRAS^G12D^ mutation in CTOs. For the first dataset, cells were seeded in BME and cultured as described above. Treatments (DMSO or RMC-9945) were added 2 days after seeding, and MTOs were collected at 48 hours, with treatment refresh at 24 hours. RNA was extracted using the PureLink RNA Mini Kit (Life Technologies, 12183025) and PureLink DNAse (Life Technologies, 12185010) following the manufacturer’s specifications. RNA was eluted using distilled water (Invitrogen, 10977-035), and the concentration was determined using the Nanodrop 1000 spectrophotometer (Thermo Fisher Scientific). Libraries were prepared using RNA-seq Stranded mRNA Library Illumina and sequenced with Illumina NextSeq 2000 P3. Trim Galore (v0.0.6, RRID: SCR_011847) was utilized for quality control and adapter trimming of FASTQ files. Single-end RNA-seq reads were aligned to either the *Mus musculus* mm10 genome (for MTO and CTO samples) or the *Homo sapiens* hg38 genome (for PDO samples) using STAR (v2.7.10a) with default parameters. Gene-level count matrices were generated using the featureCounts function from the Rsubread package (RRID: SCR_016945, based on the appropriate GENCODE (RRID: SCR_014966) primary assembly GTF annotations: vM25/Ensembl 100 for mouse and v34 for human samples. Subsequent differential gene expression analysis was conducted using DESeq2 (v1.42.0).

### scRNA-seq

FACS-sorted EPCAM^+^/CD45^−^ epithelial cells were concentrated by centrifugation (500 *g* for 10 minutes at 4°C) and counted. Cells were partitioned into gel bead in emulsions with a target of 10,000 cells per sample using the Chromium Controller system (10X Genomics). cDNA sequencing libraries were prepared using the Chromium Single Cell 3′ Kit v3 (PN-1000075). cDNA was amplified, quality checked, and quantified on an Agilent Bioanalyzer High Sensitivity chip (Agilent Technologies) and then used for gene expression library preparation (PN-220103 Chromiumi7 Sample Index Plate). Final libraries were indexed by PCR and verified for size and concentration. Sequencing was performed on an Illumina NovaSeq 6000 to obtain approximately 40,000 reads per cell.

### scRNAseq Data Analysis

Cellular transcriptome was profiled using 10X Genomics CellRanger software (v.8.0.1, RRID: SCR_017344). Reads were mapped to the mouse reference transcriptome, mm10 (GENCODE vM23/Ensembl 98, RRID: SCR_014966) in which the EGFP sequence was incorporated to track its expression. All the analyses were conducted in R (v4.3.1). Quality control of the raw data included checking the number of genes, unique molecular identifiers, and the percentage of mitochondrial RNA per cell. Filtering of low-quality cells was performed by calculating the median absolute deviation (MAD) for the number of features (nFeatures) and the number of reads (nCounts) per sample. Only cells within a maximum threshold of 5 × MAD, a minimum of 200 nFeatures, and a mitochondrial RNA percentage below 20% were included in the analysis. Additionally, ribosomal genes and the *Malat1* gene were discarded to prevent their high expression from influencing the normalization of the rest of the genes. After quality control, we preprocessed the data using the Seurat package (v5.0.2). This process involved normalizing gene expression measurements for each cell using the SCTransform function (SCT method, v2) while regressing out the percentage of mitochondrial reads to mitigate their potential influence. To eliminate nonepithelial cells from the sorted population, we assessed the expression levels of albumin, protein tyrosine phosphatase receptor type, familial adenomatous polyposis, alpha-actin-2, and platelet endothelial cell adhesion molecule. Furthermore, the positive expression of epithelial cell adhesion molecule (EpCAM) and EGFP enabled the retention of epithelial malignant cells for downstream analysis. Next, we identified highly variable features, performed principal component analysis, and scaled the data. Following this, clustering analysis was conducted using Seurat’s standard workflow. This involved constructing a shared nearest neighbor graph and applying the Louvain algorithm to identify clusters of cells with similar expression profiles. The resolution parameter was iteratively tuned to achieve an optimal balance between the number of clusters and their biological relevance. To visualize the final clusters, we used Uniform Manifold Approximation and Projection (UMAP). Differentially expressed genes for each cluster were identified using Seurat’s FindAllMarkers function with the Wilcoxon rank-sum test, retaining only those with a corrected *P* value ≤0.05. UMAP plots illustrate the spatial distribution of annotated cell types, whereas feature plots highlight the expression patterns of key marker genes. Additionally, the Markov Affinity-based Graph Imputation of Cells (MAGIC) method (v.2.0.3; ref. 63) was applied for denoising and imputing missing values, thereby restoring the data structure of our epithelial cells. Finally, the AUCell method (v1.28.0; 64) was used to define expression scores for gene signatures.

### Gene Expression Signatures

For *Lgr5*^+^ ISCs, we used the gene signature of *Lgr5*-EGFP^hi^ cells described by Muñoz and colleagues (29). The EpiHR poor prognosis signature was described by Cañellas-Socias and colleagues (13), regenerative stem cells by Vasquez and colleagues (15), fetal gut organoids by Mustata and colleagues (28), and basal PDAC in ref. (37). The rest correspond to standard Hallmark and Kyoto Encyclopedia of Genes and Genomes pathways or Gene Ontology Biological Processes as indicated. The composition of each gene signature is detailed in Supplementary Table S2.

### SLAM Sequencing

For SLAM sequencing, MTOs were pretreated 48 hours after seeding with DMSO or 100 nmol/L of RMC-9945 for 30 minutes, 4, 8, or 12 hours. Newly synthesized RNA was labeled for 120 minutes at a final concentration of 400 μmol/L with 4-thiouridine (Carbosynth). After media removal, MTOs were collected with 1 mL TRIzol (Life Technologies, 15596026) and the lysate was snap-frozen. RNA was extracted using TRIzol reagent followed by chloroform phase separation. Total RNA was subjected to alkylation by iodoacetamide (Sigma, 10 mmol/L) for 15 minutes, and RNA was repurified by ethanol precipitation. A total of 500 ng alkylated RNA were used as input for generating 3′-end mRNA sequencing libraries using a commercially available kit (QuantSeq 3′ mRNA-Seq Library Prep Kit FWD for Illumina and PCR Add-on Kit for Illumina, Lexogen). Deep sequencing was performed using NovaSeqX platforms (Illumina).

### Primary Analysis of SLAM-Seq Data

#### 3′ Untranslated Regions

Gene and 3′ untranslated region (UTR) annotations were assembled as described in Muhar and colleagues (41). In this study, annotations were obtained from the UCSC table browser (https://genome.ucsc.edu/cgi-bin/hgTables, June 2016). 3′ UTR annotations were assigned to Entrez GeneIDs and collapsed on a per-gene basis using bedtools’ merge command. For genes lacking an annotated 3' UTR, Ensembl v84 3′ UTRs were added if available. Adapters were trimmed from raw reads using cutadapt through the trim_galore (v0.3.7) wrapper tool with adapter overlaps set to 3bp for trimming. Trimmed reads were further processed using SlamDunk v0.2.4 (http://github.com/t-neumann/slamdunk), running the full analysis procedure (slamdunk all) and aligning against the mouse genome (mm10), trimming 12bp from the 5′ end, reporting up to 100 alignments for multimappers and activating the multi-mapper retention strategy, filtering for variants with a variant fraction of 0.2, and filtering for base-quality cutoff of ≥27. Unless indicated otherwise, reads were filtered for having ≥2 T > C conversions. The remaining parameters were left to their defaults. Genes with total counts of five or greater across all samples were retained for downstream analysis. Subsequently, differential expression analysis using the T > C matrix was performed using DESeq2 (v1.42.0) in a pairwise manner, in which each time point under RMC-9945 treatment (2.5, 6, 10, 14 hours) was compared with the corresponding control (DMSO). Gene expression data were normalized using the varianceStabilizingTransformation function to adjust for library size. To capture the temporal dynamics of gene signatures following RMC-9945 treatment, the vst matrix was scaled within each sample.

### Association between Oncogenic Alterations and the EpiHR Score

Annotations of oncogenic alterations of biologically relevant pathways for the TCGA samples were downloaded from ref. (3). A Wilcoxon test comparing the expression, scores of gene signatures between mutated and WT samples was performed independently for every alteration (65). The difference in expression median values was used as a measure of the impact of each mutation in the gene expression. Differential gene expression between mutant and WT samples was performed using the R package limma including “center” as a covariate in the linear model.

### ATAC-seq

AKTP MTOs were cultured and treated 48 hours after seeding, and treatment was renewed after 24 hours, as described above. ATAC-seq was performed following published protocols (66, 67).

#### Cell Lysis and Transposition

Dissociation was done in 1.5 mL 1:10 TrypLE medium for 30 minutes at 37°C with vigorously pipetted every 10 minutes, quenched in Advanced DMEM/F12 (Gibco, 12634028), supplemented with 10 mmol/L HEPES (Gibco, 11560496) and Glutamax (Gibco, 11574466), and quickly centrifuged for 5 minutes at 500 *g* at 4°C. The pellet was washed in cold PBS, and cells were lysed in 300 μL cold ATAC-seq resuspension buffer (RSB; 10 mmol/L Tris-HCl pH 7.4, 10 mmol/L NaCl, 3 mmol/L MgCl_2_ in water) containing 0.1% IGEPAL CA-630 (Sigma, l8896) following centrifugation for 10 minutes at 500 *g* at 4°C. A total of 200 μL of the supernatant was aspirated, and centrifugation was repeated for 5 minutes. Counting was done in RSB with a Neubauer chamber, and aliquots of 50,000 nuclei were prepared and resuspended in 50 μL transposition mix [25 μL 2x TD buffer (Illumina, 20034197), 2.5 μL transposase (Illumina, 20034197), 16.5 μL PBS, 0.5 μL 1% digitonin (Millipore, 300410), 0.5% 10% Tween-20 (Sigma-Aldrich, P9416), and 5 μL water] by pipetting up and down 10 times. Transposition reactions were incubated at 37°C for 30 minutes in a thermomixer and then cleaned up using Qiagen MinElute PCR Purification Kit (Qiagen, 28004).

#### Library Preparation for Sequencing

Libraries were prepared as previously described (66). Double-sided bead purification was done in amplified libraries with AMPure beads to recover fragments between 100 bp and 1.000 bp. Final libraries were eluted in 20 μL of water, and their concentration and profile were assessed with Qubit HS dsDNA (Invitrogen, Q33321) and Tapestation (Agilent, 5067–5589, 5067–5590, and 5067–5365, 5067–5366), respectively. Libraries were sequenced with Illumina NextSeq 2000 2 × 100 bp PE sequencing for at least 200 M reads.

#### ATAC-seq Analysis

ATAC-seq data were processed using the nf-core/atacseq pipeline (v2.1.2) with the June 2020 mouse reference genome (GRCm39/mm39), a pre-built BWA index, and default parameters, except for –deseq2_vst false. Consensus peaks were generated by intersecting MACS2-called peaks using bedtools (v2.31.1, RRID: SCR_006646), requiring a minimum of 10% overlap. These consensus peaks were used for downstream differential accessibility analysis. Heatmaps were generated with deepTools (v3.5.6, RRID: SCR_016366). Motif enrichment analysis was performed using MEME (v5.5.4, RRID: SCR_001783) on differentially accessible peaks (|log_2_ fold change| > 1), with the HOCOMOCOv11 full mouse database. Finally, TF motif hits were filtered using a q-value threshold of <0.01 and ranked by ascending enrichment score.

#### ChromBPNet Analysis

ChromBPNet is a deep learning DNA sequence model that predicts base-resolution accessibility profiles (RRID: SCR_024806). It learns and deconvolves assay-specific enzyme biases from the underlying regulatory sequence features governing chromatin accessibility. This enables identification of compact TF motif lexicons using ATAC-seq profiles as input (bioRxiv 2024.12.25.630221). ChromBPNet was applied as described in the original study to first infer the bias model based on vehicle-treated ATAC-seq samples. These learned biases were subsequently used to compute base-resolution contribution scores for both vehicle-treated and RMC-9945–treated samples. For each sample, bases with the top 10% contribution scores that overlapped genomic bins located spatially near the TSS of the genes of interest (see the *Methods* section) were retained. These high-contribution regions were then screened for TF motifs using the HOCOMOCOv11 full mouse database.

### Hi-C

AKTP MTOs were cultured and treated 48 hours after seeding, and treatment was renewed after 24 hours, as described above. *In situ* Hi-C was performed based on the previously described protocol (68).

#### Organoid Cross-linking and Dissociation

Organoids were crosslinked directly in the wells in 2 mL of freshly prepared cross-linking solution [1% (w/v) formaldehyde in PBS; Sigma, F8775]. Plates were incubated for 10 minutes at RT with gentle agitation (65 rpm). The reaction was quenched with 0.125 mol/L glycine for 5 minutes at RT, followed by 15 minutes at 4°C. Next, organoid suspensions were collected on ice, centrifuged (5 minutes, 500 *g*, 4°C), and washed twice with ice-cold PBS. Pellets were dissociated in 1.5 mL of prewarmed 1× TrypLE (Gibco, 12604013) and incubated for 15 minutes in a water bath, with vigorous pipetting every 5 minutes. The dissociation was quenched with 3 mL of warm Advanced DMEM/F12 (Gibco, 12634028), supplemented with 10mM HEPES (Gibco, 11560496) and Glutamax (Gibco, 11574466). Cell suspensions were centrifuged (5 minutes, 500 *g*, 4°C), washed twice with ice-cold PBS, flash-frozen in dry ice, and stored at −80°C until further processing.

#### Cell Lysis and Chromatin Digestion

For cell lysis, 600 μL lysis buffer [10 mmol/L Tris-HCl pH = 8, 10 mmol/L NaCl, 0.2% IGEPAL CA-630, 1× protease inhibitor complex (Roche, 11873580001) in water] was added to each pellet. Cells were incubated for 30 minutes on ice and immediately centrifuged for 5 minutes at 950 *g* at 4°C. All centrifugations throughout the protocol were done under the same conditions. Pellets were washed with 1× NEBuffer 2 (NEB, B7002S), resuspended in 190 μL 1× NEBuffer 2 and 10 μL 10% SDS, and incubated for 10 minutes at 65°C 350 rpm. SDS effect was quenched with 400 μL 1× NEBuffer 2 and 120 μL 10% Triton X-100 (Merck, 648466) for 30 minutes at 37°C 300 rpm. Nuclei were spun down, washed with 1x NEBuffer 2, and resuspended in 300 μL 1× NEBuffer 2 with 400 Units of MboI (NEB, R0147M). Chromatin was digested overnight at 37°C 300 rpm.

#### Biotinylation and Ligation

Samples were centrifuged, pellets resuspended with 300 μL reparation mix [30 μL 10× NEBuffer 2, 1.5 μL 10 mmol/L dCTP (Roche, 11969064001), 1.5 μL 10 mmol/L dTTP (Roche, 11969064001), 1.5 μL 10 mmol/L dGTP (Roche, 11969064001), 37.5 μL 0.4 mmol/L biotin-dATP (Thermo Fisher Scientific, 19524016), and 4.5 μL 50,000 U/µL Klenow (NEB, M0210M) in water] and incubated for 45 minutes at 37°C. Quenching was done for 10 minutes at 65°C, and DNA fragments were ligated with 1.2 mL ligation mix [120 μL 10× NEB T4 ligase buffer (NEB, M0202M), 100 μL 10% Triton X-100, 6 μL 20 mg/mL recombinant albumin (NEB, B9200S), and 5 μL 2,000,000 U/mL T4 DNA Ligase (NEB, M0202M) in water] followed by an overnight incubation at 16°C 300 rpm.

#### Cross-Link Reversion and Purification

Tubes were spun down, and nuclei were resuspended with 400 µL 1× NEBuffer 2 and 10 µl 10 mg/mL Rnase A (Thermo Fisher Scientific, EN0531) and incubated for 15 minutes at 37°C. De-crosslinking was done by adding 20 µL 800 U/mL proteinase K (NEB, P8107S) and incubating at 65°C 350 rpm for 6 hours. DNA purification was performed with 1× volume AMPure beads, and ligation efficiency was assessed with Tapestation.

#### Shearing and Library Preparation for Sequencing

One microgram of DNA was sheared in a Bioruptor Pico (Diagenode, B01080010) to 280 to 330 bp fragments in cycles of 20 sec ON and 60 sec OFF (usually six–eight cycles needed). Pull-down of the biotin tagged Hi-C fragments was done through binding to Dynabeads MyOne Streptavidin T1 beads (Thermo Fisher Scientific, 65601). In all the washes, biotinylated fragments were reclaimed with a magnetic rack. 20 µL Streptavidin bead suspension per sample was used and beads were washed with 400 µL Tween Wash Buffer (5 mmol/L Tris-HCl pH 7.5, 0.5 mmol/L EDTA, 1 mol/L NaCl, and 0.05% Tween) and then with 400 µL 2x Binding Buffer (BB; 10 mmol/L Tris-HCl pH 7.5, 1 mmol/L EDTA, 2 mol/L NaCl) and finally resuspended in 200 µL 2x BB per sample. Binding of DNA into beads was done by incubating 200 µL washed beads with 200 µL sonicated DNA for 30 minutes with rotation at RT. Two washes with 400 µL 1× BB and one with 100 µL 1× NEB T4 Ligase Buffer were done, and then beads were resuspended in 100 µL end repair mix (NEB, E6050L) following the manufacturer’s recommendations. Next, beads were washed twice with 400 µL 1× BB, once with 100 µL 1× NEBuffer 2, and then resuspended with 100 µL dATP attachment mix (NEB, E6053L; 10 µL 10× NEBNext dA-Tailing Reaction Buffer, 5 µL 5 U/µL Klenow-exo and water up to 100 µL). The mixture was incubated for 30 minutes at 37°C and then washed twice with 400 µL 1× BB and once with 100 µL 1× NEB T4 Ligase Buffer. Adapters were ligated with 10 µL 5× Quick Ligation Reaction Buffer (NEB, B6058S), 2.5 µL NEBNext Adapter (NEB, E6440S), and 2 µL 2,000,000 U/mL T4 DNA Ligase (NEB, M0202M) in water up 50 µL for 15 minutes at RT. Three microliter USER (NEB, E6440S) was added and incubated for 15 minutes at 37°C. Finally, beads were washed twice with 400 µL 1× BB and resuspended in 20 µL of water. All the resuspension was used for the final amplification with 5 µL UDIs (NEB, E6440S) and 25 µL NEBNext High-Fidelity 2× PCR Master Mix (NEB, M0541S). The PCR program was 30 seconds at 98°C, eight cycles of 10 seconds at 98°C, 30 seconds at 65°C, and 30 seconds at 72°C and a final extension of 5 minutes at 72°C. The supernatant with the amplified products was purified with 0.9× volume AMPure beads. Library concentration and size were assessed with Qubit HS dsDNA and Tapestation, respectively, and sequencing was done with Illumina NovaSeq 6000 2 × 150 bp paired-end sequencing for at least 1,000 M reads.

#### Hi-C Mapping, Matrix Generation, and Spatial Analysis of ATAC-Seq Data

Hi-C data were processed using an in-house pipeline based on TADbit (69). Reads were mapped using a fragment-based strategy, as previously described (70). Each end of the sequenced read was aligned to the June 2020 mouse reference genome (GRCm39/mm39). The TADbit filtering module was used to remove noninformative contacts to generate contact matrices, following established protocols (69). Hi-C–specific filters were then applied to exclude PCR duplicates, undigested fragments (extra-dangling ends), nonligated fragments (dangling ends), self-circles, and random breaks. The resulting contact matrices were normalized for sequencing depth and genomic biases using *cooler balance* with default parameters (71). Spatial analysis of ATAC-seq data was next performed using METALoci, as shown by Mota-Gómez and colleagues (bioRxiv 2022.11.18.516861). Briefly, 5 Kb-resolution Hi-C interaction maps were generated for 1 Mb regions centered on the TSS of *Emp1* (chr6:134,835,000–135,835,000) and *Lgr5* (chr10:114,920,000–115,925,000). Hi-C interactions were log10-transformed, and the top 20% of interactions were selected. These interactions were then converted into a distance matrix by taking their inverse. The Kamada–Kawai algorithm was applied to produce a 2D layout in which each node represents a bin from the Hi-C matrix. Using SciPy’s Voronoi function, each bin was assigned a Voronoi polygon, with dummy nodes added to close open regions and ensure all polygons were finite. A buffer zone (1.5 the mean interbin distance) was generated around each bin, and the overlap between the buffer and the corresponding Voronoi shape defined the final spatial occupancy per bin. This resulted in a stylized “worm-like” visualization referred to as Gaudí plots (Fig. 5). Finally, ATAC-seq signal was mapped onto the Gaudí plot by using pyBigWig to extract per-bin coverage. Genomic bins within 3.0 times the mean interbin distance of the TSS-containing bin (either *Emp1* or *Lgr5*) were used to identify open chromatin regions. These regions, informed by ChromBPNet-derived signal contributions, were then analyzed to predict likely bound TFs (see previous section in “Methods”).

### CUT&RUN

CUT&RUN was performed in biological duplicates following the published protocol (72), with minor modifications.

#### Cell Lysis and Antibody Binding

Dissociation was done under the same conditions as in ATAC-seq. Cells were washed twice in wash buffer [20 mmol/L HEPES pH 7.5, 150 mmol/L NaCl, 0.5 mmol/L spermidine (MERCK, 85558-1G), and 1× Roche Complete Protease Inhibitor EDTA-free in nuclease-free water (Roche, 05056489001)] for 3 minutes at 600 *g*, RT. Aliquots of 100,000 cells in 200 μL of wash buffer were prepared, one per reaction mix. Cells were then bound to concavalin A–coated magnetic beads (BioMagPlus, 86057-3), followed by resuspension in 200 μL antibody buffer consisting of wash buffer supplemented with 0.05% digitonin (MERCK, 300410-250MG), 0.5 mol/L EDTA, and the appropriate dilution of the antibody. The histone-targeting antibody H3K27ac (Abcam, ab177178) was diluted to 1:50, whereas the JunB (Cell Signaling Technology, 3753) and the negative control IgG (Invitrogen, 10500C) were diluted 1:100, following the manufacturers’ recommendations. Samples were incubated overnight at 4°C on a rocking platform to allow antibody binding.

#### Binding pAG-MNase and Chromatin Digestion

Beads were washed in 200 μL digitonin wash buffer [wash buffer supplemented with 0.05% digitonin (MERCK, 300410-250MG)] and then resuspended in 100 μL of the same buffer containing 3 μL of pAG-MNase (Cell Signaling Technology, 0366S). Samples were then incubated on a rocking platform for 1 hour at 4°C to allow pAG-MNase binding. Following incubation, beads were resuspended in 150 μL digitonin wash buffer and placed in an ice water bath (0°C) for 5 minutes prior to chromatin digestion. Digestion was performed by adding 3 μL 100 mmol/L CaCl_2_ and incubating for 30 minutes at 4°C. After digestion, 150 μL 2× STOP buffer [200 nmol/L NaCl, 20 nmol/L EDTA, 20 nmol/L EGTA (PanReac AppliChem, A0878,0025), 0.2% digitonin (MERCK, 300410-250MG), 100 μg/mL RNAse A (Thermo Fisher Scientific, EN0531), 50 μg/mL glycogen (Thermo Fisher Scientific, R0561), and 100 pg spike-in DNA (Cell Signaling Technology, 40366S) in nuclease-free water] was added, and samples were incubated for 10 minutes at 37°C for chromatin release. Tubes were centrifuged for 5 minutes at 16,000 *g*, 4°C, and placed on a magnetic rack. The supernatant containing the digested chromatin was separated and purified using the Qiagen MinElute PCR Purification Kit (QIAGEN, 28006). DNA was eluted in 25 μL of nuclease-free water, quantified with Qubit HS dsDNA (Thermo Fisher Scientific, Q33231), and fragment size distribution was assessed using a D5000 ScreenTape for TapeStation systems (Agilent, 5067–5588).

#### Library Preparation

Sequencing libraries were prepared following NEBNext Ultra II DNA Library Prep Kit (NEB, E7645L) without size selection before PCR amplification. Amplification was performed with 20 μL Q5 Master Mix, 5 μL Unique Dual Index Primer Pairs (NEB, E6440S), and 20 μL of library. The PCR program was the following: 45 seconds at 98°C, 14 cycles consisting of 15 seconds at 98°C, and 10 seconds at 60°C and a final extension of 1 minute at 72°C. PCR purification was done in 1.1× volume AMPure XP beads (BECKMAN COULTER, 082A63881). Library concentration and size were assessed with Qubit HS dsDNA (Thermo Fisher Scientific, Q33231) and TapeStation (Agilent, 5067–5588), respectively, and sequencing was done with Illumina NextSeq500 2 × 75 bp PE sequencing for at least 20 M reads per replicate.

#### CUT&RUN Preprocessing and Peak Calling

CUT&RUN reads were processed using the nf-core/cutandrun pipeline v3.2.1 (73) aligning to the *Mus musculus* reference genome (GRCm39/mm39, June 2020) and the *Saccharomyces cerevisiae* iGenome R-64-1-1 for the spike-in normalization. Spike-in reads were used to normalize signal across samples. The pipeline was executed with default parameters, and peak calling was performed using MACS2, with narrow peaks.

### Statistics

No statistical test was used to determine sample size upfront. Instead, sample size was determined empirically according to previous knowledge of the variation in the experimental set-up (27). A minimum of four mice were quantified in each experiment and for each condition. Automated blind quantifications and blind data analysis were performed whenever possible. No data from *in vivo* experiments were excluded, except for mice that were excluded if liver metastasis did not present proper engraftment 4 days after intrasplenic injections tracked with *in vivo* bioluminescence. Then, animals were randomized and treated as indicated in each experiment.

For *in vitro* experiments, we used *n* = 3 or *n* = 6 according to previous experience with similar experiments. Statistical analyses were performed using GraphPad Prism (v10.2.3) unless otherwise stated. All significant *P* values (*P* < 0.05) calculated are shown in each figure.

## Supplementary Material

Supplementary Table S1 Gene signatures used in Figure 1 (G, I, K), in Supplementary Figure S2, for the GSEA analysis in Figure 2 (B, G, J, M), in Supplementary Figure S5, in Figure 3 (A, B, C).

Supplementary Table S2 Supplementary Table S2. Hallmark, GOCC, KEGG and custom gene signatures of AKTP MTO, AKP CTO, PDO131, and PDO C31M. Selection related with Figure 2 (B, G, J, M)

Supplementary Table S3 Supplementary Table S3. Gene expression related to Supplementary Figure S6B, C, G, H, I, J

Supplementary Table S4 PDO mutations

Supplementary Table S5 qRT-PCR probes

Supplementary Figures S1-S14 Supplementary Figures S1-S14 related to the manuscript "A plastic EMP1^+^ to LGR5^+^ cell state conversion as a bypass to KRAS-G12D pharmacological inhibition in metastatic colorectal cancer"

Supplementary Movie S1 Time-lapse imaging of representative AKTP MTO organoids from vehicle and RMC-9945 treatment conditions. Initial imaging (time 0h) was performed prior to vehicle or RMC-9945 treatment. Upon treatment, organoids were imaged every hour over a 48-hour period. Top (vehicle) and bottom (RMC-9945) panels show XY planes positioned at three z-depths relative to the centre of the organoids volume (-10 µm, 0 µm and +10 µm). Lgr5-EGFP is shown in green and Emp1-TOM in red. Scale bar, 50 µm.

## Data Availability

RNA-seq data for AKTP MTO and APK CTO are available at GEO under the accession code GSE294825, whereas for PDO131 and PDO C31M, RNA-seq data are available under the accession code GSE294823. The RMC-9945 and MRTX1133 comparison data are available under the accession code GSE303692. Count matrices for scRNA-seq experiments were deposited at GEO under the accession code GSE294820. Hi-C and ATAC-seq data are available at GEO under the accession codes GSE294819 and GSE294817, respectively. CUT&RUN sequencing results are available under the accession code GSE304518. SLAM-seq data are available at GEO under the accession code GSE294822. Computer code is available upon request. Further information and requests for resources, biological material (MTOs), and reagents should be directed to the corresponding author.
