# Supplementary material for: A Plastic EMP1+ to LGR5+ Cell State Conversion as a Bypass to KRASG12D Pharmacologic Inhibition in Metastatic Colorectal Cancer
Source: Cancer Discov. 2025 Oct 21;16(2):320–44. doi: 10.1158/2159-8290.CD-25-0679 (PMC12877754; doi:10.1158/2159-8290.CD-25-0679)
Supplement: Supplementary Figures S1-S14 — related to the manuscript "A plastic EMP1+ to LGR5+ cell state conversion as a bypass to KRAS-G12D pharmacological inhibition in metastatic colorectal cancer" [file cd-25-0679_supplementary_figures_s1-s14_suppsf1.pdf]

Supplementary Figure S1

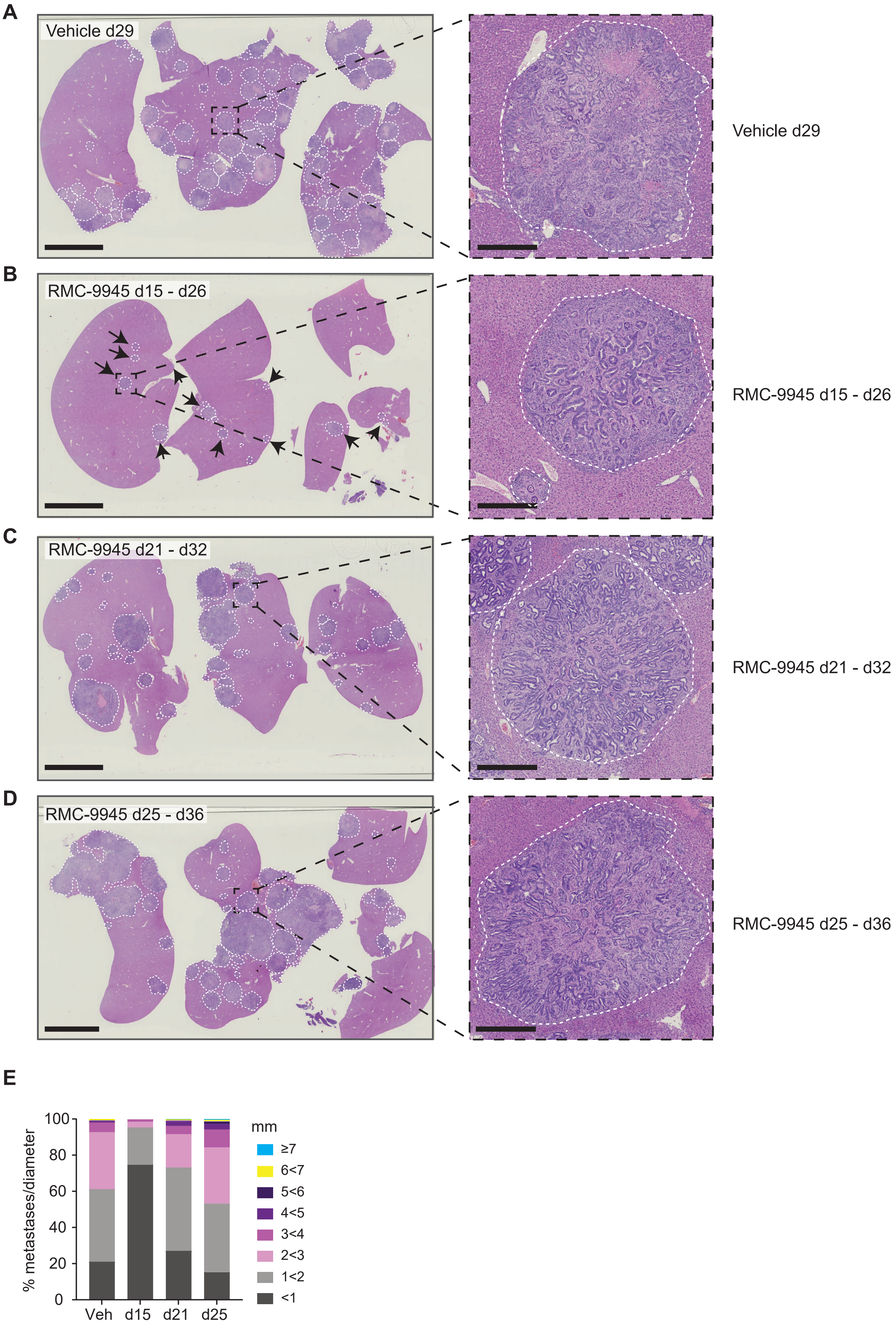

Supplementary Figure S1. Histology and metastasis size upon RMC-9945 treatment. A-D, Representative H/E images of livers treated with vehicle (**A**) or RMC-9945 starting at different days post inoculation. **B**, 15 days, **C**, 21 days, **D**, 25 days. Arrows pointing at liver metastases. Dashed lines depicting individual metastases. Magnification of individual metastases showed in the right panels. Left panels scale bar, 5 mm. Right panels scale bar, 500 µm. **E**, Quantification of the proportion of metastases per size (higher diameter). Vehicle n=5 mice, d15 n=7, d21 n=8, d25=8.

Supplementary Figure S2

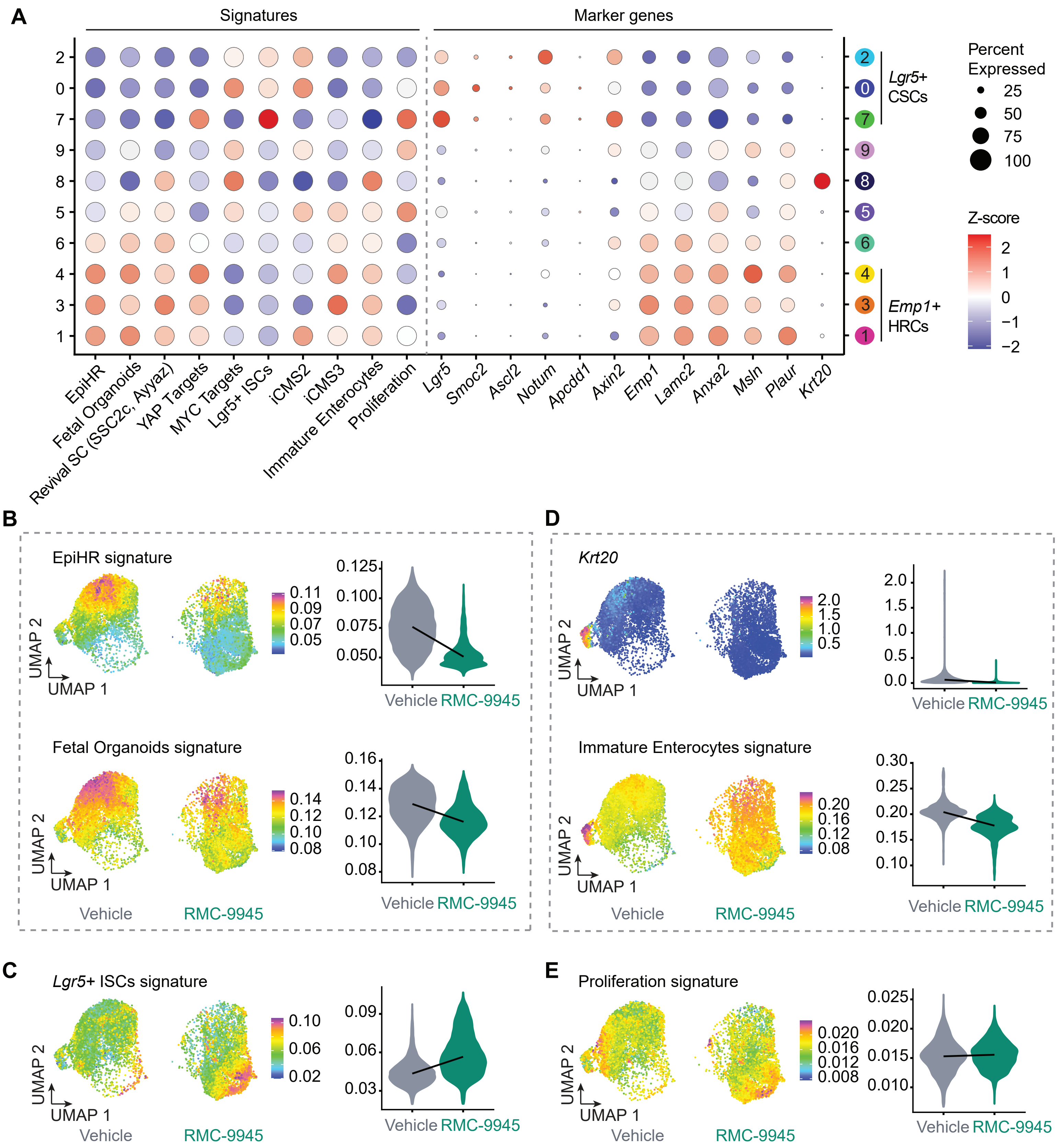

Supplementary Figure S2. **Cell state marker genes and signatures in AKTP liver metastases. A**, Dot plot showing the scaled UCell scores for selected gene signatures (dot size is not meaningful in this context) and normalized SCT expression for individual genes across clusters (Louvain clustering, resolution = 0.6). B-E, UMAP visualization depicting the expression of EpiHR signature (13), Fetal organoids signature (28)(**B**), *Lgr5*+ ISC signature (29) (**C**), *Krt20* mRNA expression along with Immature Enterocytes (77) (**D**), and crypt proliferation gene signatures (**E**)(MAGIC values) stratified by vehicle and RMC-9945 treatment. Corresponding violin plots highlight expression distribution patterns and median differences. Gene signatures used for analysis are detailed in **Supplementary Table 1**.

Supplementary Figure S3

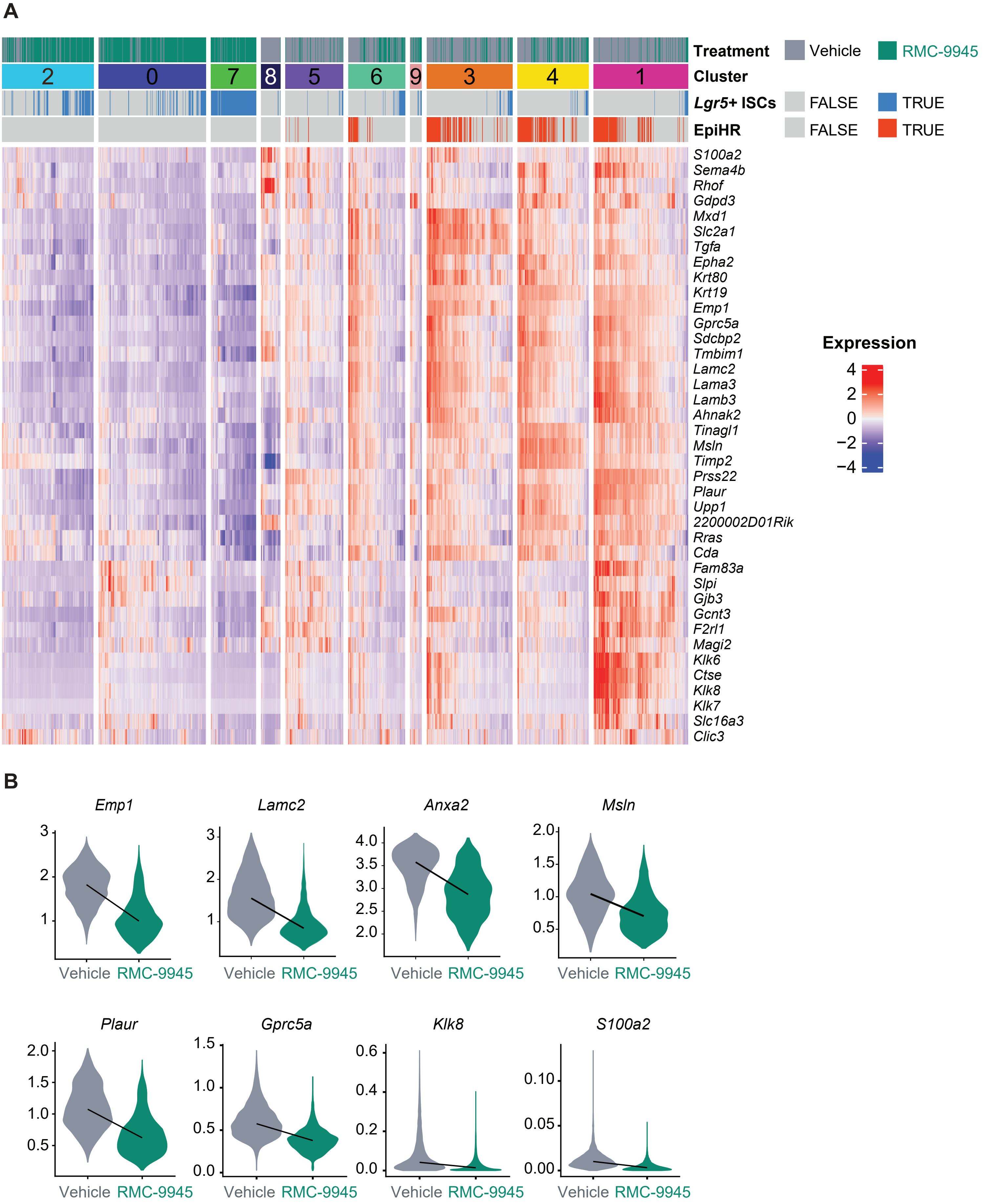

Supplementary Figure S3. Expression of the poor prognosis EpiHR signature (Cañellas-Socias et al.) and markers genes in AKTP liver metastases. A, Heatmap of single cells from liver metastases, hierarchically clustered using the Ward D2 method based on EpiHR plus HRC core gene expression (13)(MAGIC values, GSEA leading edge selection). Columns represent individual cells, color-coded by treatment, cluster membership (Louvain clustering, resolution = 0.6), and *Lgr5*+ ISC or HRC membership.

B, Violin plots comparing EpiHR-related gene expression between vehicle (gray) and RMC-9945 (green) conditions, highlighting distribution patterns and median differences.

Supplementary Figure S4

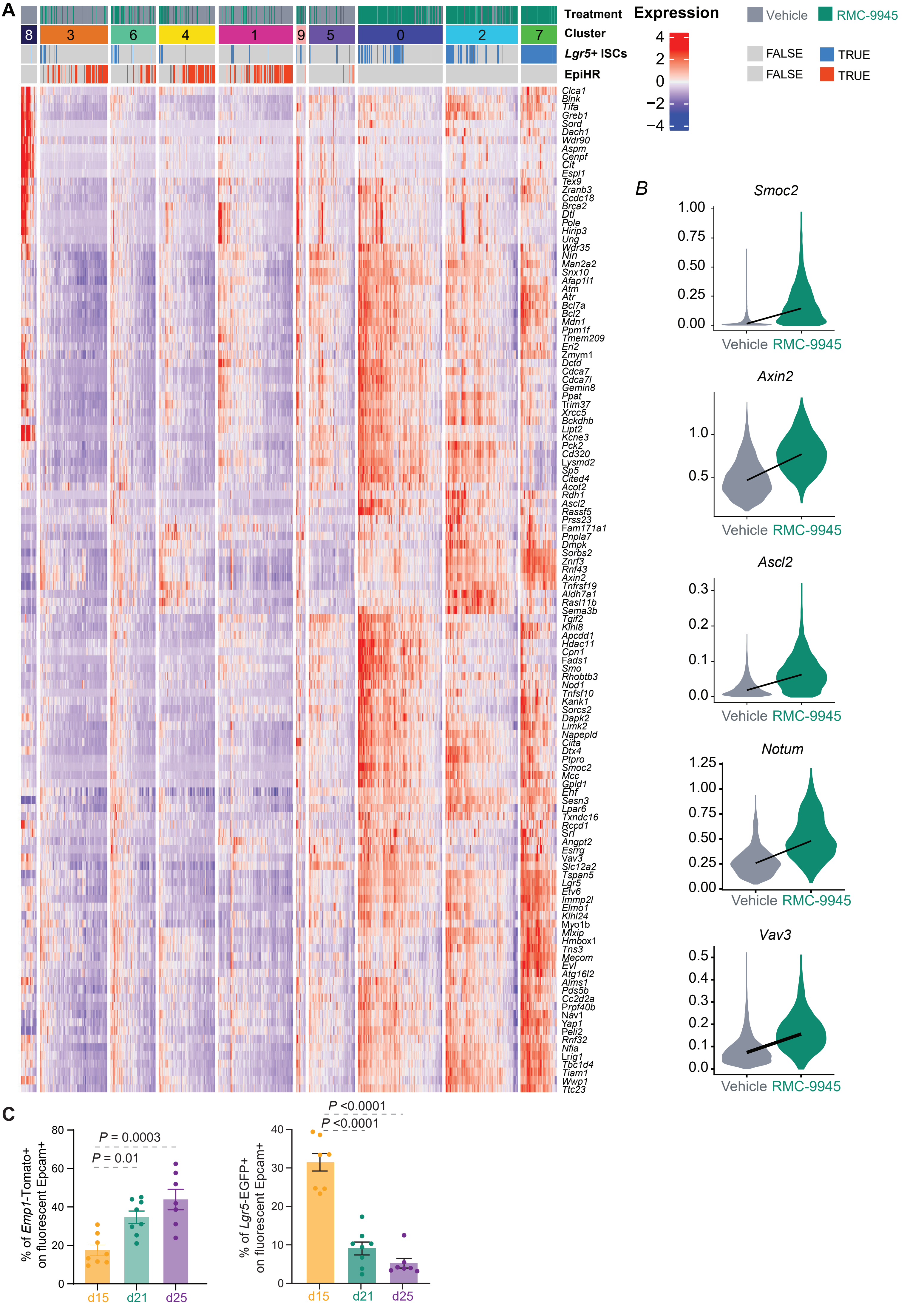

Supplementary Figure S4. Expression of *Lgr5*+ ISC signature (Muñoz et al) and markers genes in AKPT liver metastases. A, Heatmap of single cells from liver metastases, hierarchically clustered using the Ward D2 method based on *Lgr5*+ ISC gene signature (29) (MAGIC values, GSEA leading edge selection). Columns represent individual cells, color-coded by treatment, cluster membership (Louvain clustering, resolution = 0.6), and *Lgr5*+ ISC or HRC membership. **B**, Violin plots comparing *Lgr5*+ ISC-related gene expression between vehicle (gray) and RMC-9945 (green) conditions, highlighting distribution patterns and median differences. **C**, FACS quantification of the percentage of *Emp1*-TdTomato+ and *Lgr5*-EGFP+ cells within the fluorescent-positive EPCAM+ population of disaggregated liver metastasis derived from *Emp1*-iCasp9-tdTomato and *Lgr5*-EGFP AKTP MTOs at 15 (n=8), 21 (n=8) and 25 (n=7) days after instraspleen injection. Mean ± SEM; P values are derived from ANOVA followed by Tukey's test.

Supplementary Figure S5

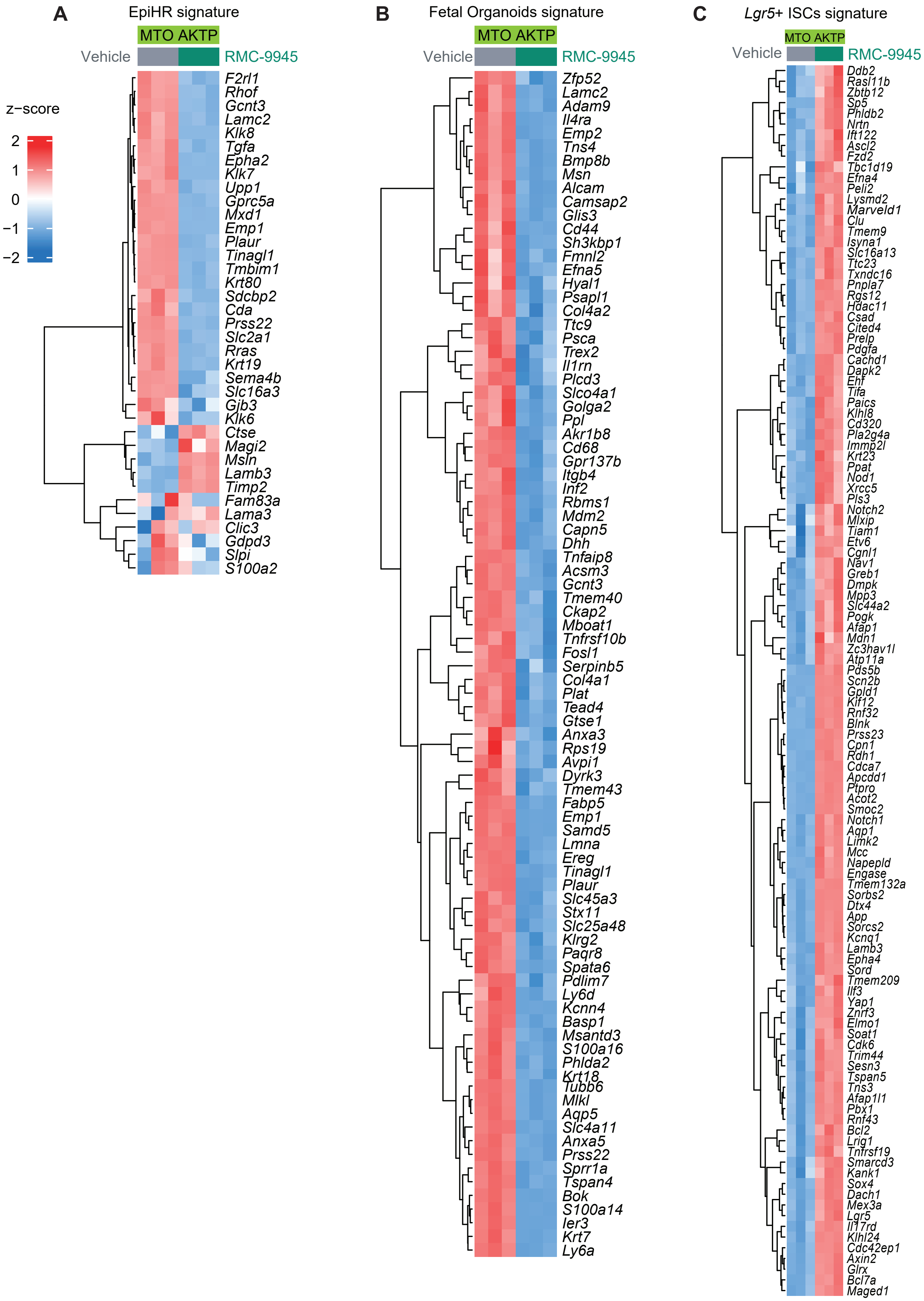

Supplementary Figure S5. Expression of *Lgr5*+ ISC, EpiHR and Fetal organoids signatures in AKTP MTOs. **A-C**, Heatmap of *in vitro* AKTP organoids treated with vehicle or RMC-9945, hierarchically clustered using the Ward D2 method based on *Lgr5*+ ISC - (**A**), along with EpiHR (**B**) and Fetal Organoid (**C**) gene signatures (MAGIC values, GSEA leading edge selection).

Supplementary Figure S6

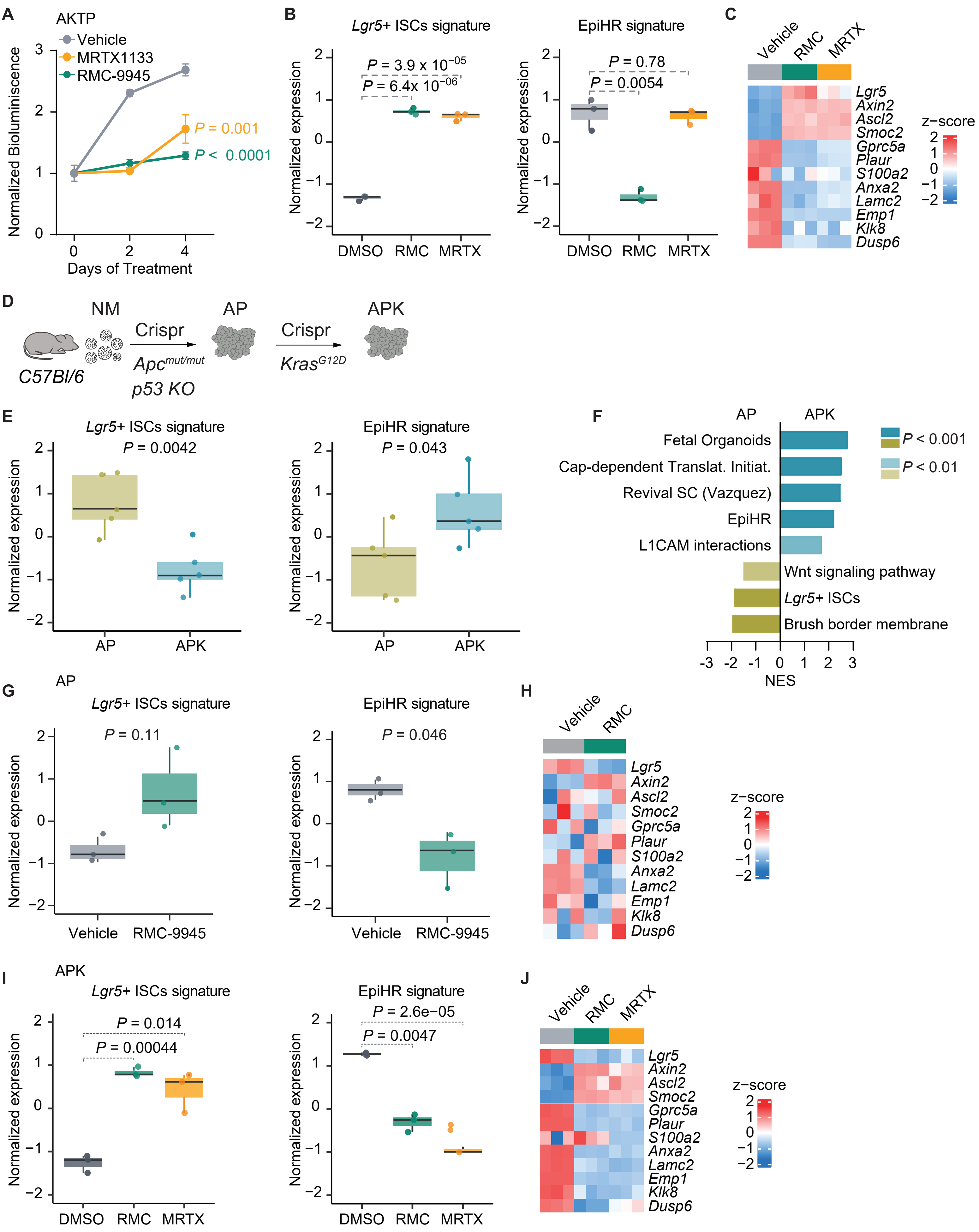

Supplementary Figure S6. ***Emp1*+ HRC to *Lgr5*+ CSC states plasticity by oncogenic KRAS**. **A**, Growth (CellTiter-Glo) of AKTP MTOs treated with vehicle, RMC-9945 or MRTX-1133 for 4 days normalized to day 0 of treatment (n=6 per group). Mean ± SEM; P values are derived from ANOVA followed by Dunnett´s test. **B**, Boxplots depicting the normalized and z-scored expression of *Lgr5*+ ISC gene signature and EpiHR signature in AKTP CTOs treated with either vehicle or RMC-9945 for 48 hours. T-test was used to assess median differences. **C**, Heatmap showing expression of selected ISC-related and EpiHR genes across treatment conditions in AKTP organoids. n=3 per treatment. **D**, Schematic representation of CRISPR-based generation of tumor organoids. Organoids derived from normal intestinal mucosa (NM, C57BL/6) were engineered using CRISPR to inactivate Apc and Trp53 (AP organoids), followed by introduction of Kras G12D to generate APK organoids. **E**, Boxplots depicting the normalized and z-scored expression of *Lgr5*+ ISC gene signature and EpiHR signature in AP versus APK mouse tumor organoids; statistical comparisons were performed using two-tailed t-tests and P values are indicated. **F**, Gene set enrichment analysis (GSEA) of signatures and pathways enriched in AP versus APK organoids, including *Lgr5*+ ISC and EpiHR programs, Wnt signaling, and translation initiation pathways. **G**, Boxplots depicting the normalized and z-scored expression of *Lgr5*+ ISC gene signature and EpiHR signature in AP organoids tumor upon RMC-9945; statistical comparisons were performed using two-tailed t-tests and P values are indicated. **H**, Heatmap showing expression of selected ISC-related and EpiHR genes across RMC-9945 treatment conditions in AP tumor organoids. **I,** Boxplots depicting the normalized and z-scored expression of *Lgr5*+ ISC gene signature and EpiHR signature; statistical comparisons were performed using two-tailed t-tests and P values are indicated. **J**, Heatmap showing expression of selected ISC-related and EpiHR genes across treatment conditions in APK organoids.

Supplementary Figure S7

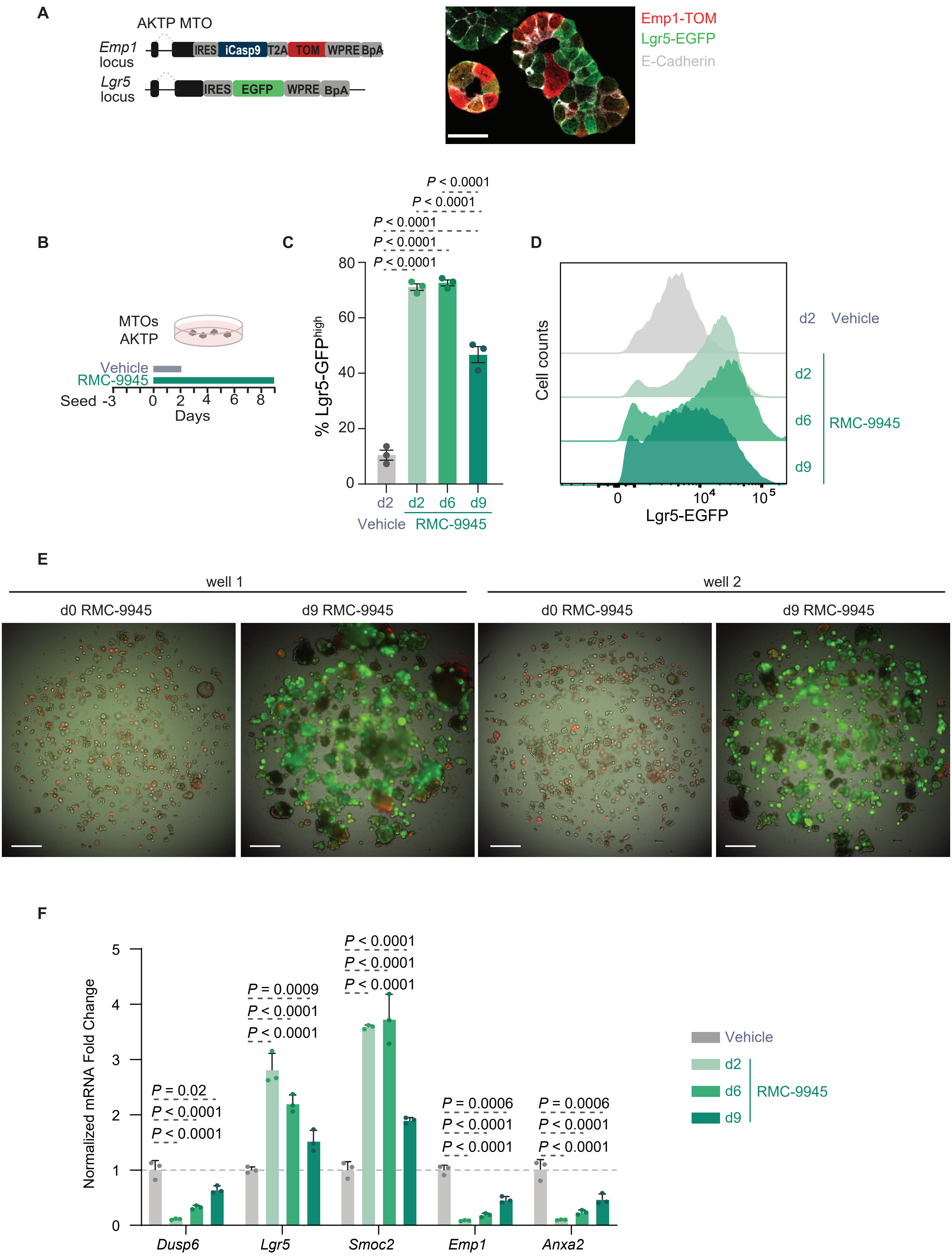

Supplementary Figure S7. Expression of the ISCs program upon continuous RMC-9945 treatment. A, *Emp1*-iCasp9-tdTomato and *Lgr5*-EGFP alleles introduced in AKTP MTOs. Confocal imaging of TOM, EGFP and E-Cadherin immunostaining in CRISPR knock-in MTOs. Single z-plane. Scale bar, 30 µm. This panel is shown in Figure 4A. **B**, Schematic of *in vitro* AKTP MTOs treated with vehicle for 2 days or RMC-9945 for 9 days, with daily media change. **C,** FACS quantification of the percentage of *Lgr5*-EGFP+ cells from AKTP CRISPR knock-in organoids treated with vehicle for 2 days or RMC-9945 for 2, 6 or 9 days. Mean ± SEM; P values are derived from ANOVA followed by Tukey's test. **D,** Representative histograms of *Lgr5*-EGFP expression in vehicle and RMC-9945 AKTP MTOs at different time points. **E,** Brightfield 4x magnification microscopy images of AKTP *Emp1*-TOM/*Lgr5*-EGFP MTOs treated with at day0 and treated with RMC-9945 for 9 days. Scale bar: 500 µm. Two different wells are shown. **F**, RT-qPCR analysis of the expression of the indicated genes in AKTP organoids treated with vehicle for 2 days or with RMC-9945 for 2, 6 and 9 days. Mean ± SEM; P values are derived from ANOVA followed by Tukey's test.

Supplementary Figure S8

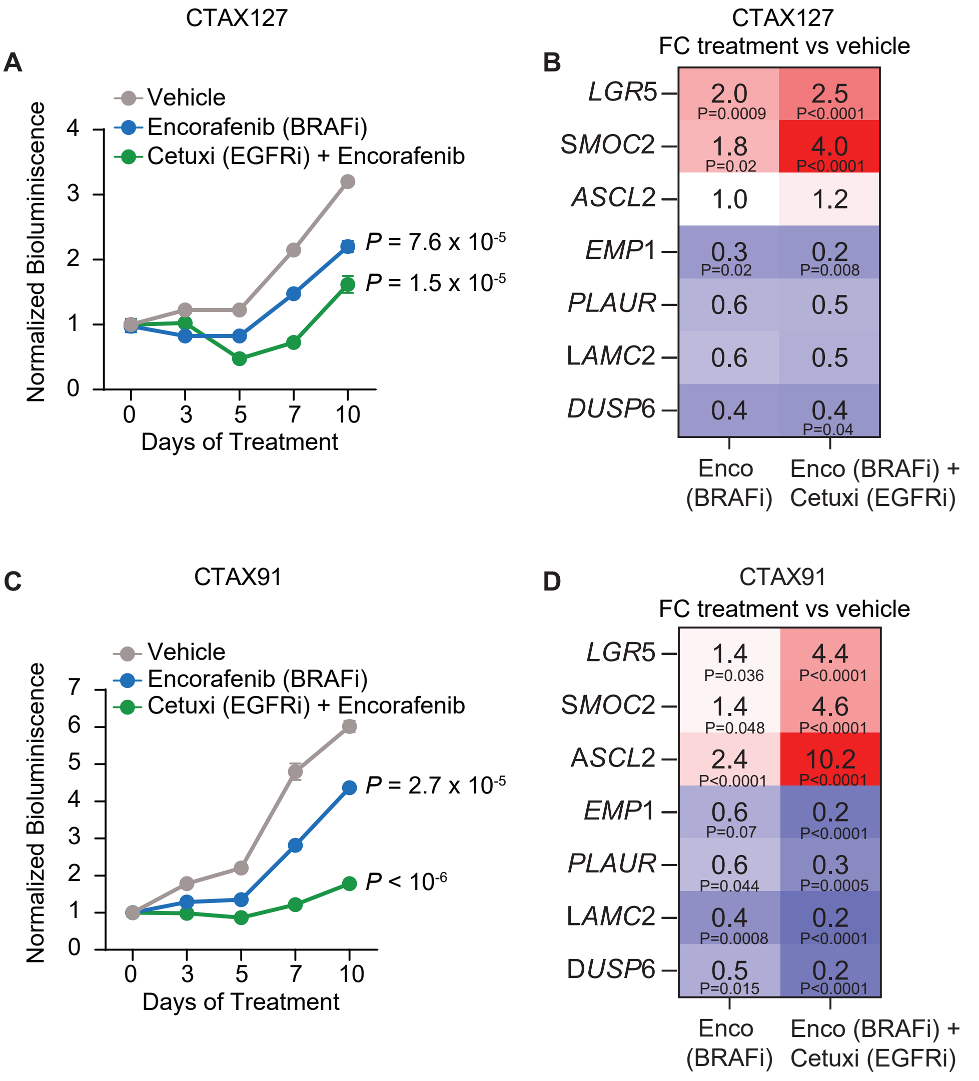

Supplementary Figure S8. Plasticity induced by BRAF V600E inhibition. A, B, Growth (CellTiter-Glo) of BRAF-V600E CTAX127 (**A**) and heatmap of fold change gene expression for indicated genes (**B**). **C, D,** Growth (CellTiter-Glo) of BRAF-V600E CTAX91 (**C**) and heatmap of fold change gene expression for indicated genes (**D**). For growth analysis CTAX127 and CTAX91 organoids are treated with vehicle, Encorafenib, Encorafenib+Cetuximab for 10 days and the data are normalized to day 0 of treatment (n=5 per group). Mean ± SEM; P values are derived from ANOVA followed by Welch t-test. For gene expression analysis organoids are treated for 24 hours with vehicle, Encorafenib or Encorafenib+Cetuximab. P values are derived from ANOVA followed by Tukey’s test.

Supplementary Figure S9

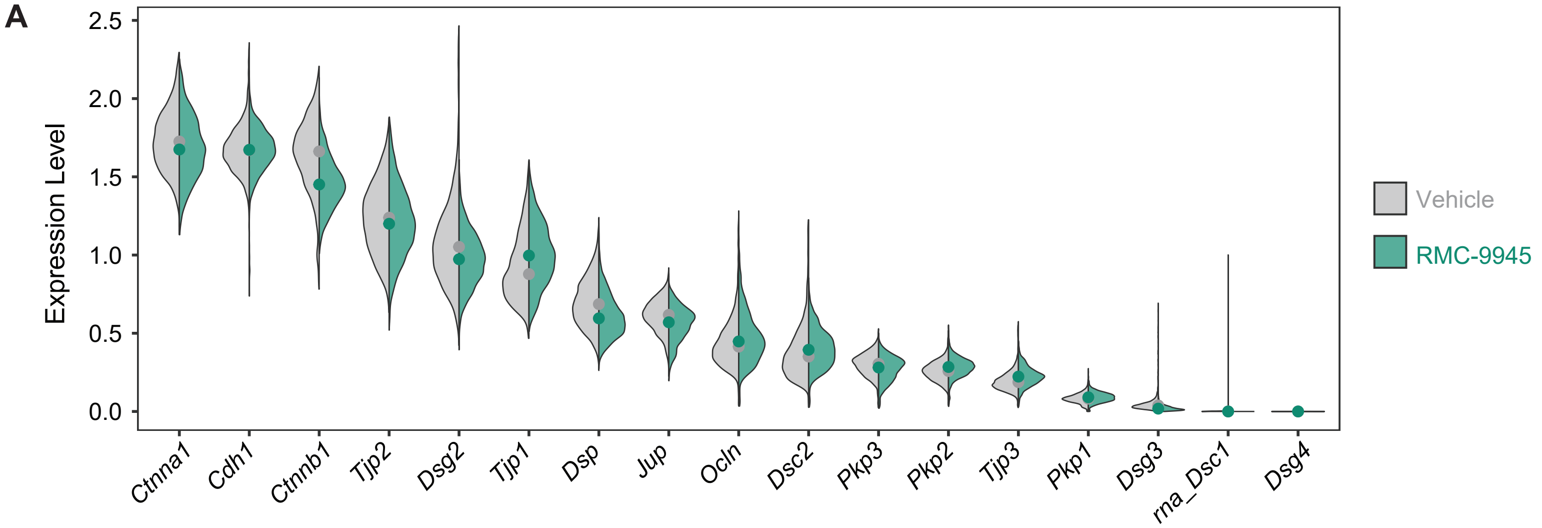

B

	PDO C31M		PDO 131	
**GENE**	**Log2 FC**	**P_adj**	**Log2 FC**	**P_adj**
KRT6A	-2,00	3,1E-05	-0,49	1,9E-01
KRT7	-2,55	8,6E-07	-0,48	5,5E-01
KRT6B	-1,41	3,4E-06	-0,60	2,3E-01
KRT8	-0,26	3,6E-09	-1,30	2,9E-182
CDX2	0,61	1,5E-24	-0,13	6,0E-02
CDX1	0,30	3,6E-06	0,05	7,0E-01
ASCL2	1,37	1,07E-60	0,44	3,7E-09
HNF4G	0,70	3,7E-18	1,69	7,4E-08

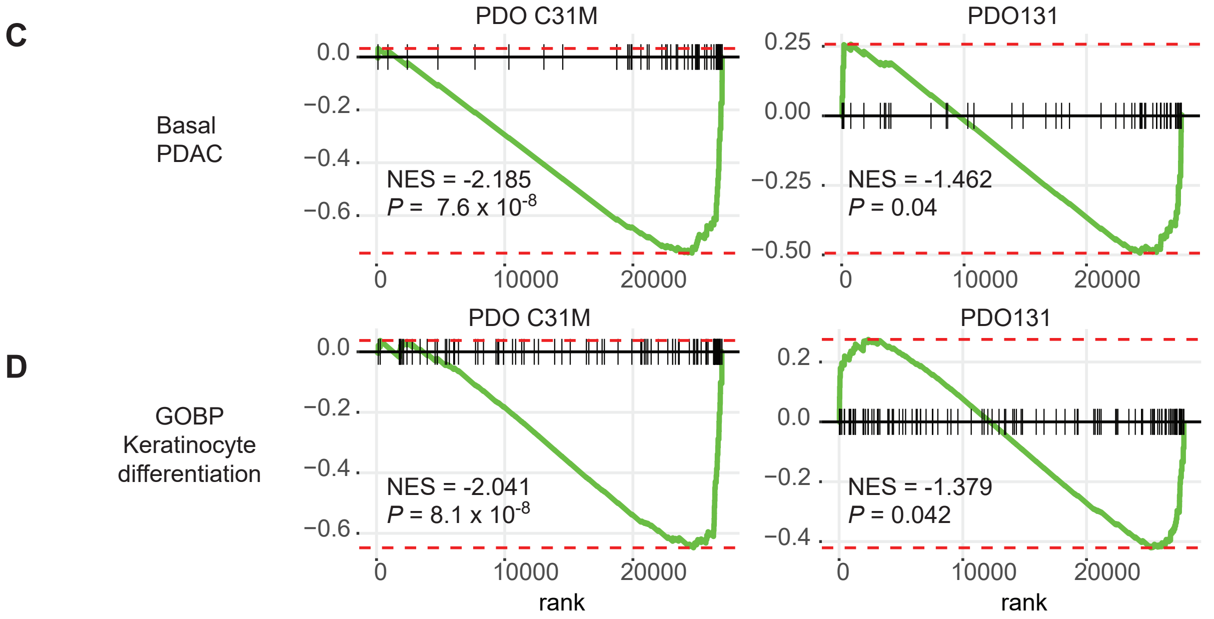

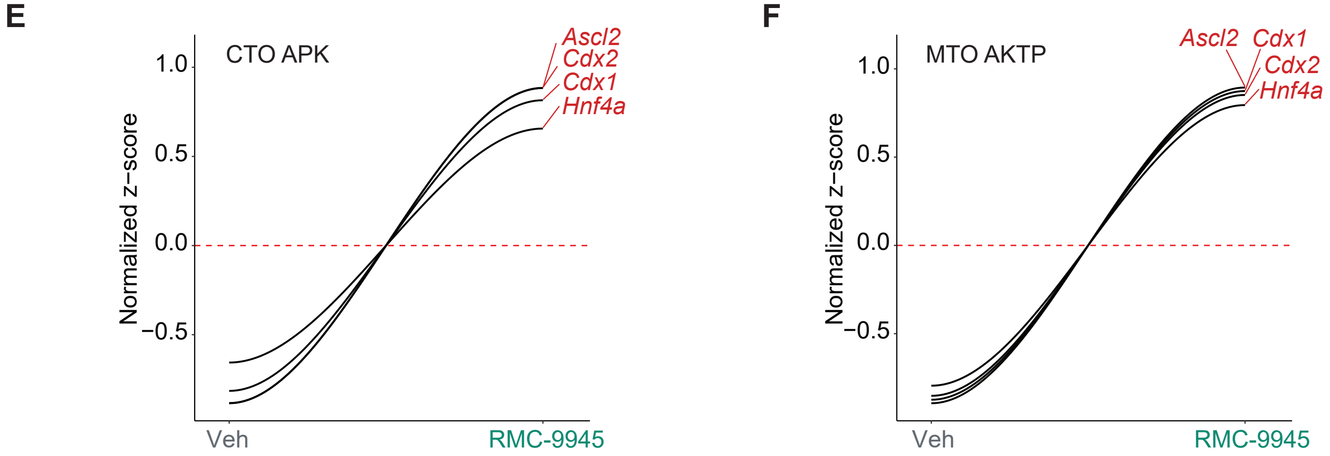

Supplementary Figure S9. Changes in cell adhesion, basal keratins and intestinal defining TFs induced by RMC-9945. A, Split violin plots showing the expression of selected genes across cells, comparing vehicle (gray) and RMC-9945 (green) conditions. Dots represent the median expression for each group. **B,** Differential gene expression analysis of keratinization and intestinal-defining genes in PDO C31M and PDO131. Log2 FC and adjusted P-value are shown. **C**, **D**, GSEA for Basal PDAC (**C**) and Keratinocyte differentiation (**D**) genes in PDO C31M, and PDO131. NES, Normalized Enrichment Score. P-values are derived from permutation testing to assess the significance of enrichment scores calculated based on gene ranking. **E**, **F**, Line chart showing normalized and scaled expression of canonical ISC genes, comparing vehicle (gray) and RMC-9945 (green) conditions in (**E**) APK and (**F**) AKTP models.

Supplementary Figure S10

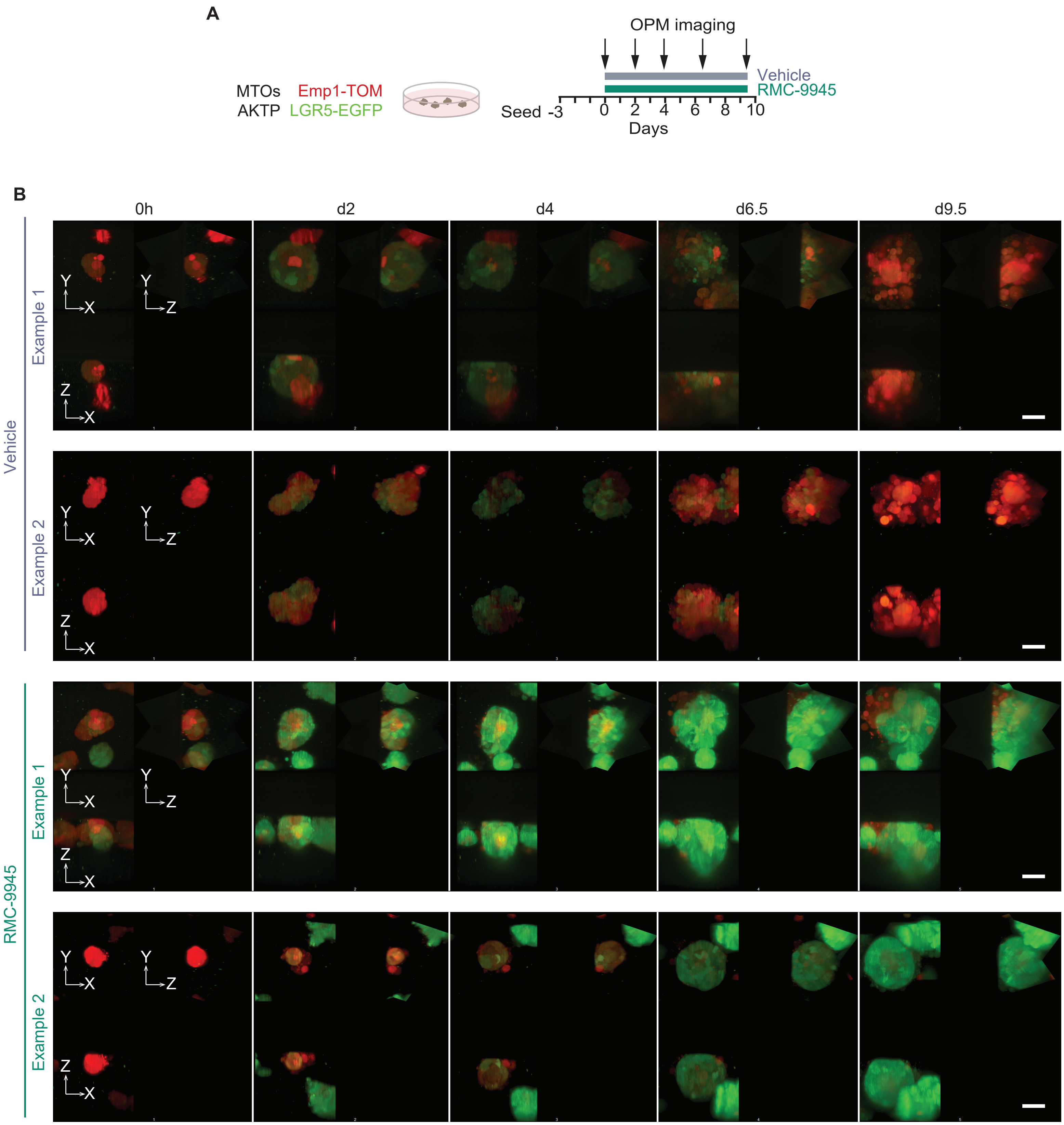

Supplementary Figure S10. Live imaging of AKTP MTOs treated with RMC-9945. A, Schematic of in vitro AKTP MTOs treated with vehicle for or RMC-9945 for 9 days. **B**, Representative orthogonal-plane maximum intensity projection images of AKTP MTOs from vehicle-treated (top two panels) and RMC-9945-treated (bottom two panels) groups. Panels show Lgr5-EGFP (green) and Emp1-TOM (red) organoids captured at 0h, d2 (50h), d4 (92h), d6.5 (160h) and d9.5 (232h). All images were displayed with identical gamma correction (γ = 0.5). Scale bar, 50μm.

Supplementary Figure S11

A

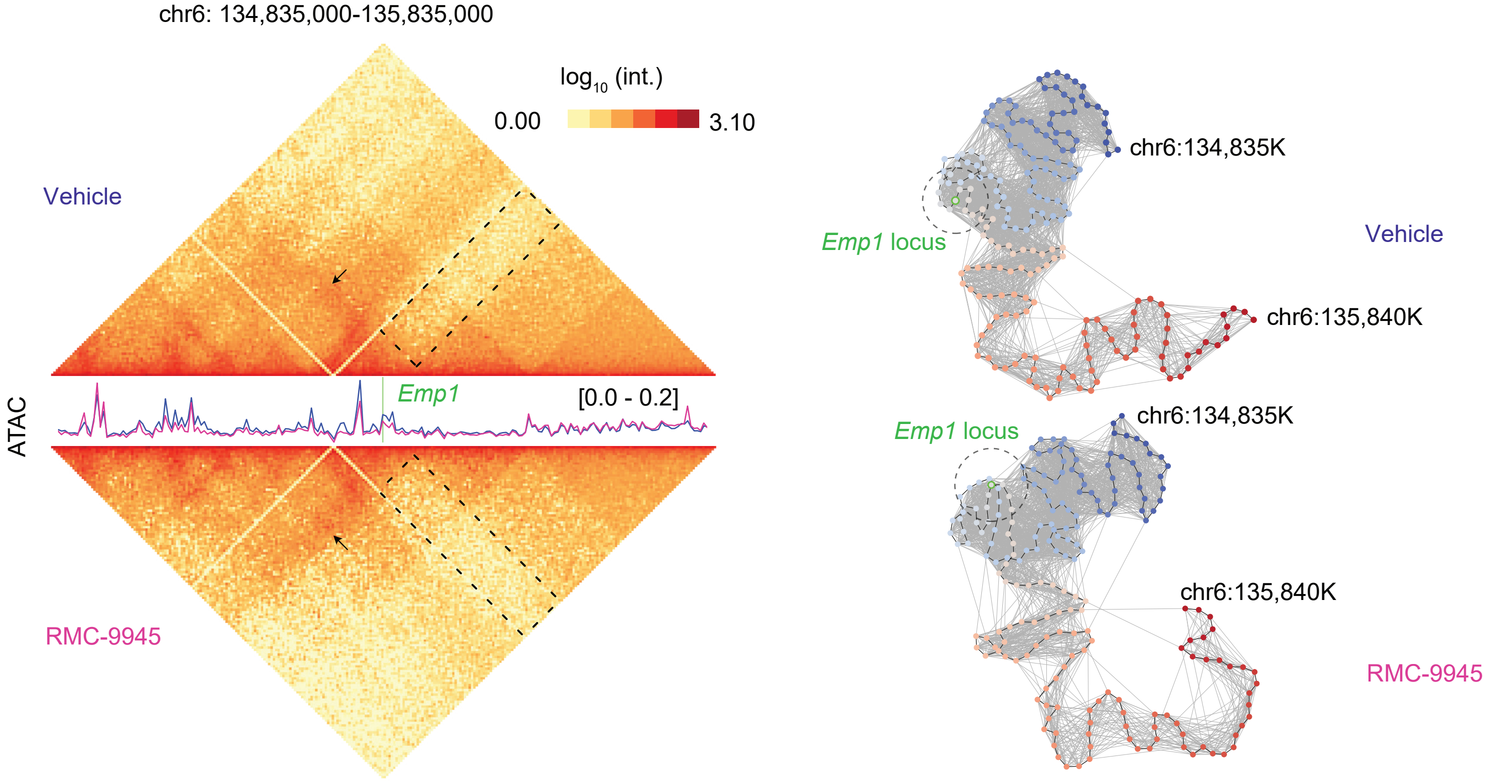

B

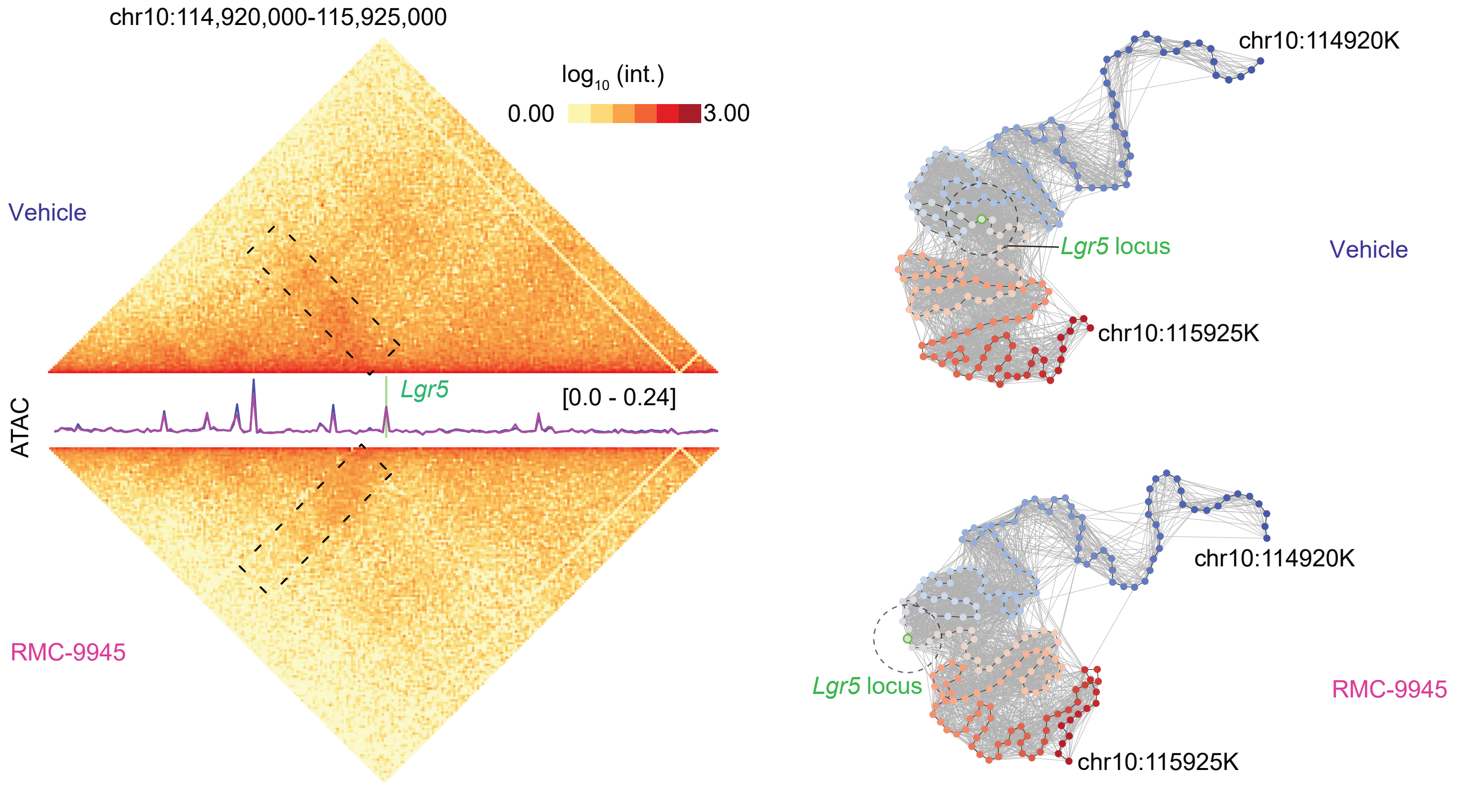

Supplementary Figure S11. Spatial (HiC) analysis of *Emp1* and *Lgr5* genomic regions. A, METALoci analysis for *Emp1* gene locus. Left. Hi-C data and ATAC-seq signal for vehicle and RMC-9945 are displayed centered at chr6:134,835,000-135,835,000. The position of the *Emp1* promoter (green vertical line) is highlighted. A region with decreased interactions in RMC-9945 treated is highlighted within a dashed rectangle. Increased loop interaction in RMC-9945 treated is marked by an arrow. Although the topological organization of the *Emp1* locus is maintained, the highlighted changes of interactions result in a modest conformational change. Right. Kamada-Kawai 2D layout highlighting the *Emp1* locus (green circle) and all genomic bins taken into consideration for the motif search (dashed circle). **B**, METALoci analysis for the *Lgr5* gene locus centered at chr10:114,920,000-115,925,000. Same representation as in **A**. A region with increased interactions in RMC-9945 treated is highlighted within a dashed rectangle. The topological organization of the *Lgr5* locus is maintained modestly affected by the increased interactions.

Supplementary Figure S12

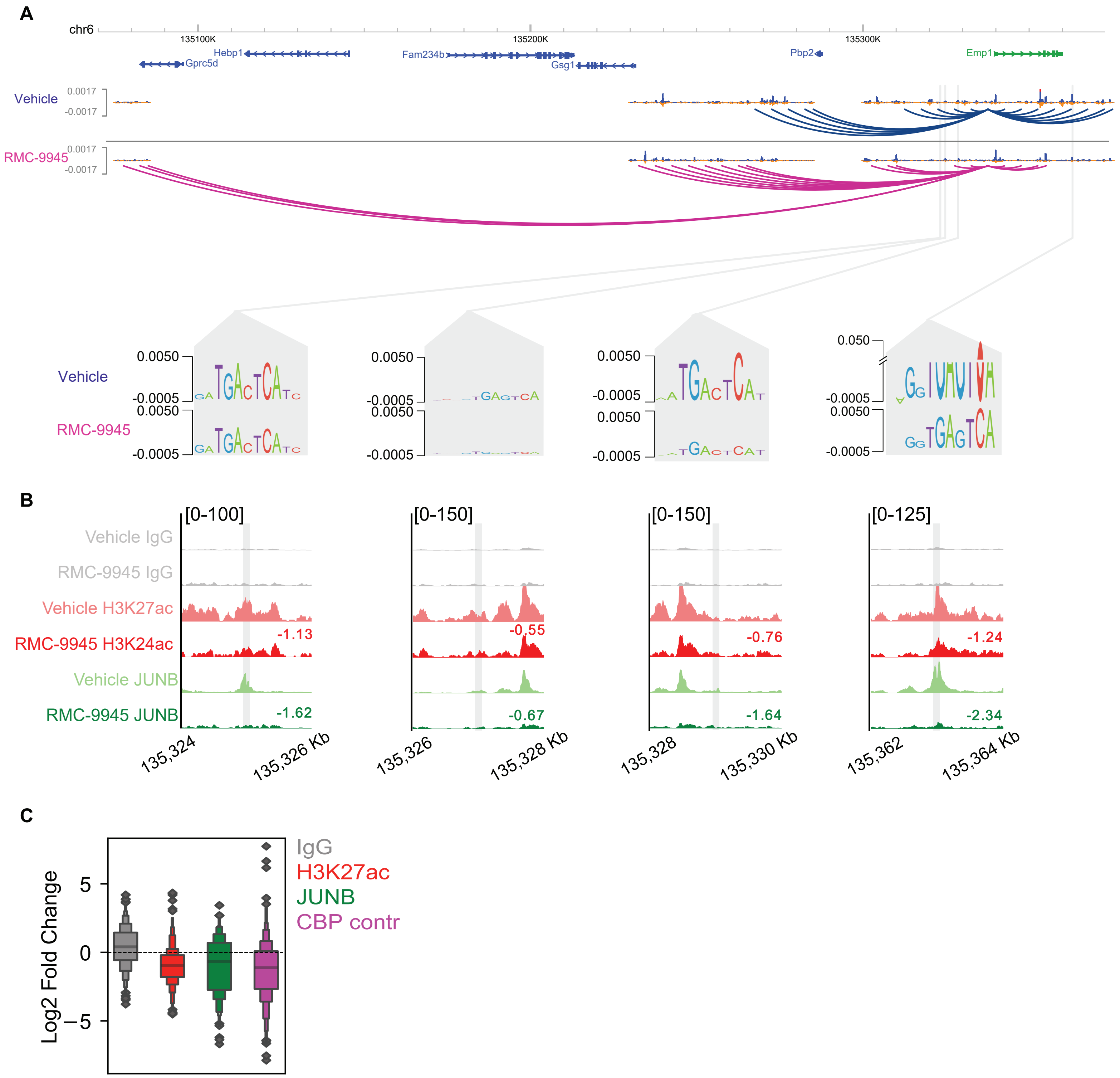

Supplementary Figure S12. AP1 binding motifs accessibility following RMC-9945 treatment. CUT&RUN validation of AP1 binding around *Emp1* gene. **A**, (Top) Genomic track covering all METALoci regions interacting either in vehicle and RMC-9945 treated as observed in the Gaudi plots in Figure 5. Arcs represent the interaction between the TSS containing 5 kbp bin and those in spatial proximity in the Gaudí plot. (Bottom) ChromBPNet contributions of the nine detected signatures for AP-1 in the interacting regions for both Vehicle and RMC-9945. **B**, CUT&RUN tracks for 2kb regions around the motif site. The tracks show IgG, H3K27ac and JUNB tracks. Numbers in RMC-9945 H3K27ac and RMC-9945 JUNB tracks show the in log2(FoldChange) of RMC-9945 with respect Vehicle. **C**, Log2(FoldChange) of each mark observed in a total of 385 motifs identified in EpiHR genes. All for distributions are statistically different to zero as for one-sample t-test (all distributions have p-value < 10e-5).

Supplementary Figure S13

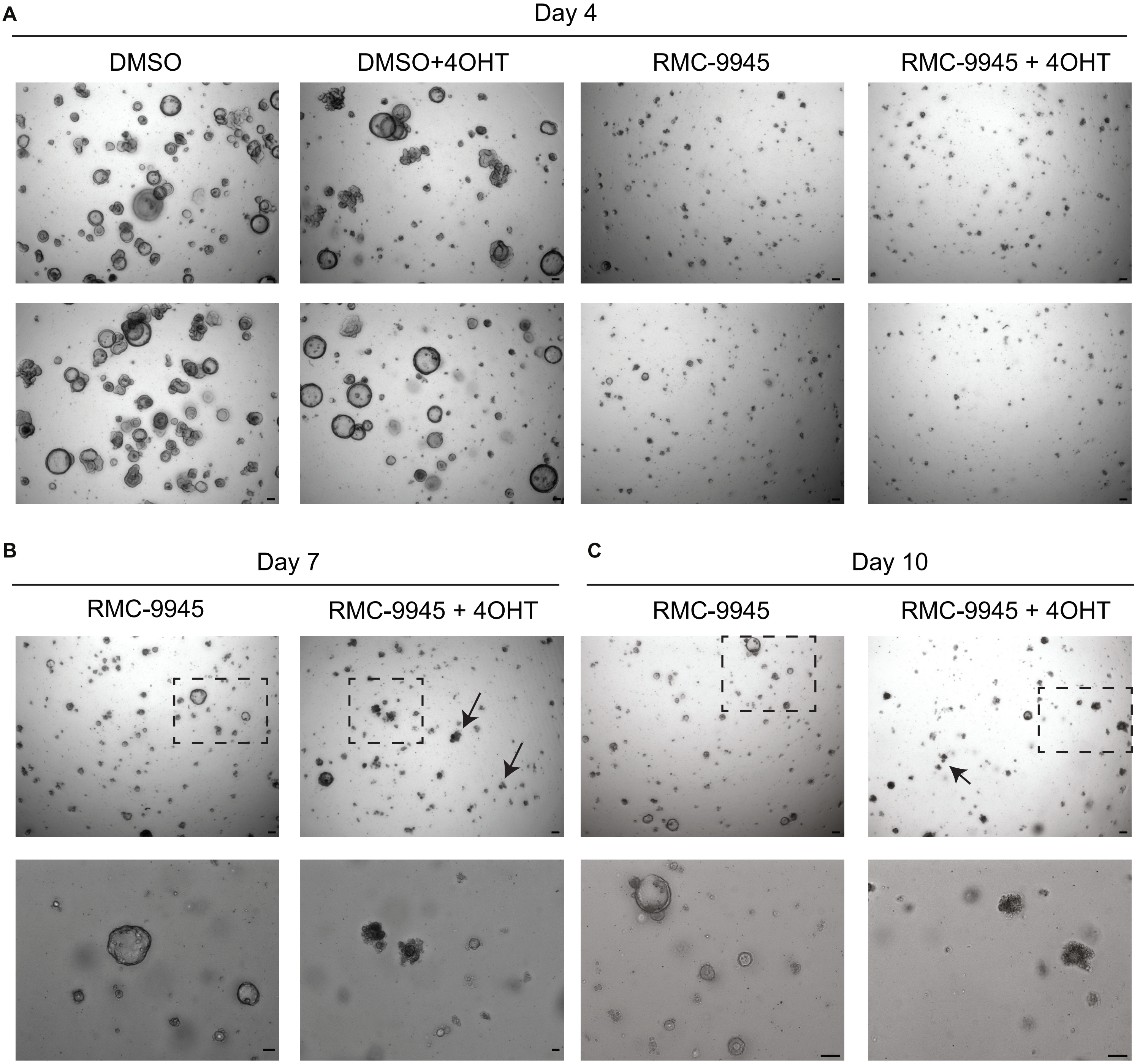

Supplementary Figure S13. RMC-9945 increases β-catenin/TCF transcriptional activity. A, Bright Field 4x magnification microscopy images of AKTP NTCF-ERT2 *Emp1*-TOM/*Lgr5*-EGFP MTOs treated with vehicle, RMC-9945, 4-OH-tamoxifen+vehicle or 4-OH-tamoxifen+RMC-9945. Scale bar: 100 μm. **B, C,** Bright Field 4x (top panel) and 10x (bottom panel) magnification microscopy images of AKTP NTCF-ERT2 *Emp1*-TOM/*Lgr5*-EGFP MTOs treated with RMC-9945 or 4-OH-tamoxifen+RMC-9945 for 7 days (**B**) or 10 days (**C**). Arrows point to necrotic cells. Scale bar: 100 μm.

Supplementary Figure S14

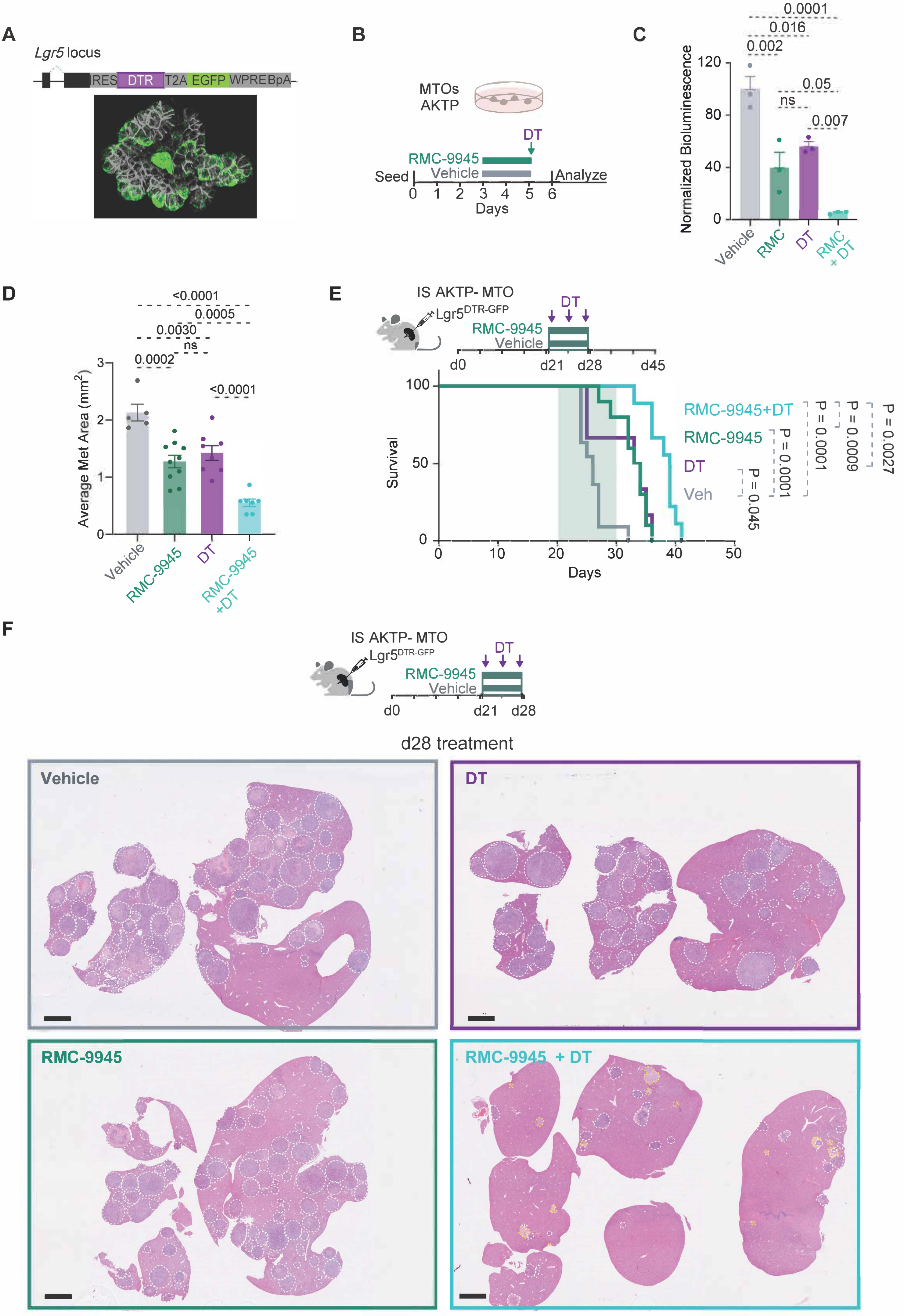

Supplementary Figure S14. Dependency on *Lgr5*⁺ CSCs in RMC-9945 treated metastases. A, CRISPR-Cas9 targeting strategy to introduce a DTR-GFP cassette into the *Lgr5* locus of MTOs. Confocal imaging of immunostaining for EGFP and EPCAM in *Lgr5*-DTR-EGFP organoids. Scale bar, 30 µm. **B**, Schematic of AKTP MTOs treated with vehicle or RMC-9945 for 48h, with one administration of DT 24h before analysis, and **C**, Percentage of alive cells (luminescence) measured with CellTiter-Glo. P values are derived from ANOVA followed by Tukey's tests. **D**, Quantifications of the average metastatic area (mm^2). Vehicle n=5 mice, RMC-9945 n=10, DT n=8, RMC-9945+DT=7. Mean ± SEM. P values are derived from ANOVA followed by Tukey's tests. **E**, Kaplan-Meier survival plot of mice treated with the indicated treatments. P values are derived from Gehan-Breslow-Wilcoxon test. **F**, Representative H&E images of livers from AKTP MTO instraspleen-inoculated mice treated with vehicle, RMC-9945, DT or RMC-9945+DT from day 21 to day 28 post-injection.

Supplementary Data

Supplementary Table 1. Gene signatures used in Figure 1 (G, I, K), in Supplementary Figure S2, for the GSEA analysis in Figure 2 (B, G, J, M), in Supplementary Figure S5, in Figure 3 (A, B, C).

Supplementary Table 2. Hallmark, GOCC, KEGG and custom gene signatures of AKTP MTO, APK CTO, PDO131, and PDO C31M. Selection related to Figure 2 (B, G, J, M).

Supplementary Table 3. Differential expression analysis of AKTP MTO, AP and APK CTOs treated with vehicle, RMC-9945 or MRTX1133.

Supplementary Table 4. Mutations identified in the PDO and PDXO models described.

Supplementary Table 5. Probes used for qRT-PCR analysis.

Supplementary Movie 1. Time-lapse imaging of representative AKTP MTO organoids from vehicle and RMC-9945 treatment conditions. Initial imaging (time 0h) was performed prior to vehicle or RMC-9945 treatment. Upon treatment, organoids were imaged every hour over a 48-hour period. Top (vehicle) and bottom (RMC-9945) panels show XY planes positioned at three z-depths relative to the centre of the organoids volume (-10 µm, 0 µm and +10 µm). L*gr5*-EGFP is shown in green and *Emp1*-TOM in red. Scale bar, 50 µm.
